# Switching Ion Binding Selectivity of Thiacalix[4]arene Monocrowns at Liquid–Liquid and 2D-Confined Interfaces

**DOI:** 10.3390/ijms22073535

**Published:** 2021-03-29

**Authors:** Anton Muravev, Ayrat Yakupov, Tatiana Gerasimova, Ramil Nugmanov, Ekaterina Trushina, Olga Babaeva, Guliya Nizameeva, Viktor Syakaev, Sergey Katsyuba, Sofiya Selektor, Svetlana Solovieva, Igor Antipin

**Affiliations:** 1 FRC Kazan Scientific Center, Arbuzov Institute of Organic and Physical Chemistry, Russian Academy of Sciences, Arbuzov Str. 8, 420088 Kazan, Russia; gryaznovat@iopc.ru (T.G.); olbazanova@iopc.ru (O.B.); vsyakaev@iopc.ru (V.S.); katsyuba@iopc.ru (S.K.); 2 Butlerov Institute of Chemistry, Kazan Federal University, Kremlevskaya Str. 18, 420008 Kazan, Russia; yakupov.airat21@mail.ru (A.Y.); RaINugmanov@kpfu.ru (R.N.); svsol@iopc.ru (S.S.); iantipin54@yandex.ru (I.A.); 3 School of Chemistry, University of Glasgow, University Avenue, Glasgow G12 8QQ, UK; kate1809@mail.ru; 4 Department of Physics, Kazan National Research Technological University, Karl Marx Str. 68, 420015 Kazan, Russia; guliya.riv@gmail.com; 5 Frumkin Institute of Physical Chemistry and Electrochemistry, Russian Academy of Sciences, Leninsky pr. 31, 119071 Moscow, Russia; sofs@list.ru

**Keywords:** thiacalix[4]arene monocrowns, Langmuir monolayers, liquid-phase extraction, ion binding, surface potential, UV/visible reflection–absorption spectroscopy

## Abstract

Understanding the interaction of ions with organic receptors in confined space is of fundamental importance and could advance nanoelectronics and sensor design. In this work, metal ion complexation of conformationally varied thiacalix[4]monocrowns bearing lower-rim hydroxy (type I), dodecyloxy (type II), or methoxy (type III) fragments was evaluated. At the liquid–liquid interface, alkylated thiacalixcrowns-5(6) selectively extract alkali metal ions according to the induced-fit concept, whereas crown-4 receptors were ineffective due to distortion of the crown-ether cavity, as predicted by quantum-chemical calculations. In type-I ligands, alkali-metal ion extraction by the solvent-accessible crown-ether cavity was prevented, which resulted in competitive Ag^+^ extraction by sulfide bridges. Surprisingly, amphiphilic type-I/II conjugates moderately extracted other metal ions, which was attributed to calixarene aggregation in salt aqueous phase and supported by dynamic light scattering measurements. Cation–monolayer interactions at the air–water interface were monitored by surface pressure/potential measurements and UV/visible reflection–absorption spectroscopy. Topology-varied selectivity was evidenced, towards Sr^2+^ (crown-4), K^+^ (crown-5), and Ag^+^ (crown-6) in type-I receptors and Na^+^ (crown-4), Ca^2+^ (crown-5), and Cs^+^ (crown-6) in type-II receptors. Nuclear magnetic resonance and electronic absorption spectroscopy revealed exocyclic coordination in type-I ligands and cation–π interactions in type-II ligands.

## 1. Introduction

Ion binding is of fundamental and practical interest in coordination and supramolecular chemistry, because it involves a multitude of weak interactions and requires an induced fit with the receptor [1,2]. In particular, these features are decisive for efficiency of receptor units pre-organized on a calixarene scaffold [3,4]. Calixarenes have been exploited as ion receptors in liquid-phase extraction, fluorometric sensing, self-assembly on solid surfaces, and coordination network formation [5,6,7,8]. Lower-rim modification of calixarenes with oxyethylene units has provided calixarene–crown-ether conjugates (calixcrowns), which display exceptional metal ion binding efficiency and selectivity [9].

Outstanding receptor characteristics of calixcrowns under liquid-phase extraction conditions and structural effects of the macrocycles on binding selectivity were extensively investigated. Selectivity of calix[4]crown-ethers is mainly governed by size complementarity, according to which the crown-4 unit is well suited for Li^+^, while crown-5 and crown-6 units prefer K^+^ and Cs^+^, respectively [10,11]. Insofar as monomers are considered, an increase in the number of crown-ether units affects marginally ion extractability of calixcrowns [12]. There is a more complex interplay for steric factors, such as calixarene conformation, upper-rim substitution pattern, and macrocycle cavity size (comparison of calixarenes and thiacalixarenes) [9,13,14]. Such interplay originates from the stabilizing effect of inverted aryl rings in *partial cone* and *1,3-alternate* configurations of calixarene due to ion–π interactions and the destabilizing steric factor from bulky groups at upper/lower rims, the extent of which inversely depends on the calixarene and crown-ether cavity sizes.

**Figure 1:**
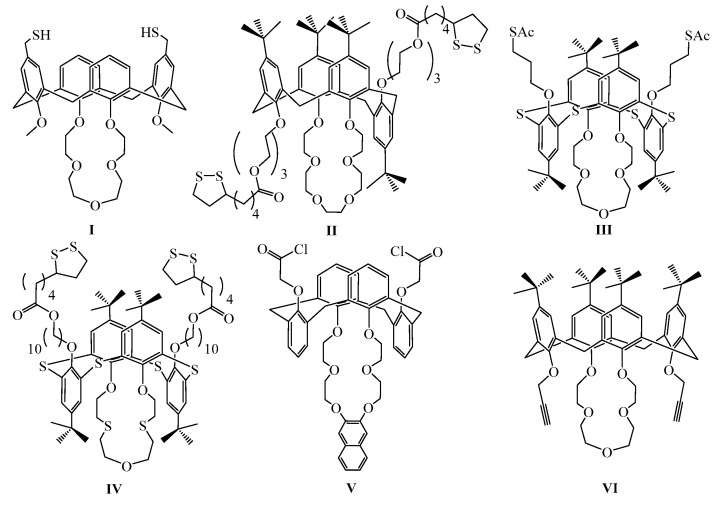
Examples of heterotopic calixcrown-ether receptors with anchoring groups [15,16,17,18,19,20].

More diverse applications of ion-coordinating ability were demonstrated in confined space for calixcrowns functionalized by anchoring groups (Figure 1). One example is *cone* calix[4]crown-5 **I** modified with CH_2_SH units on the upper rim, which formed self-assembled monolayers on gold and recognized polyamines [15]. Similarly, *partial cone* analog **II** with dithiolane fragments on the lower rim displayed potentiometric recognition of alkaline-earth vs. alkali metal ions (although the role of crown-ether moiety in such discrimination is not evident) [16]. A different approach was employed in our group to facilitate enzyme adhesion to gold substrate by amphiphilic *1,3-alternate* thiacalix[4]monocrown-5 **III** in Langmuir monolayers [17]. A significant advance in covalent confinement of calixcrowns was achieved through immobilization of thiacrown **IV**–Au nanoparticles onto polymer membrane to give an ultrasensitive response towards Ag^+^ [18]. The groups beyond sulfur functionalities are also used to graft calixcrowns onto the substrates bearing complementary reaction centers. One example is the Cs^+^ fluorescence probe based on crown-6 **V** linked to hybrid (NH_4_)_3_PMo_12_O_40_–silica mesoparticles via amide groups [19]. Another example is the calixcrown-ether **VI** monolayer grafted onto silica with a triazole spacer, which demonstrated K^+^-controlled pesticide binding/release monitored by contact angle measurements [20].

Additional synthetic stages for the attachment of anchoring groups to calixcrowns, as well as their interference in the complexation behavior of crown-ether macrocycle, restrict the choice of metal ions and calixcrowns for the rational design of advanced materials. The Langmuir method, which pre-organizes molecules at the air–water interface and provides tightly packed monolayers under lateral compression, is a convenient tool as it does not require specific anchoring groups and could be effective in evaluating ion binding in thin-film sensors. There has been a unique study of calixcrown-ethers in Langmuir monolayers, which revealed Cs^+^/Na^+^ selectivity [21]. Intriguingly, switching of metal ion selectivity in calixarenes with the transition from liquid phase to air–water interface has been recently documented [22]. Thus, comparison of various environments is highly desirable in the study of binding behavior of these host molecules. Here, we report the complexation study of metal ions with topologically varied thiacalix[4]monocrowns at the liquid–liquid interface under extraction conditions and at the air–water interface using the Langmuir monolayer technique.

## 2. Results and Discussion

### 2.1. Design, Synthesis, and Characterization of Thiacalix[4]monocrown-Ethers

**Figure 2:**
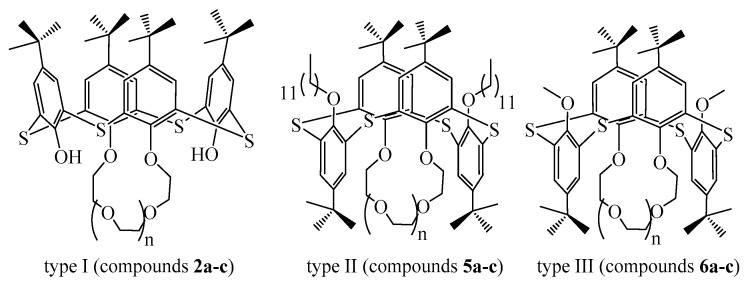
Type I–III thiacalix[4]monocrowns studied in this work (n = 4–6).

Figure 2 outlines structural variations in thiacalixcrown-ethers (conformation of macrocycle, number of O atoms in crown-ether, and alkyl chain length). Three structural types were considered:

(1) Ionizable *cone* thiacalixcrowns, with four to six oxygen atoms in the crown-ether ring (type I);
(2) Amphiphilic *1,3-alternate* thiacalixcrowns bearing C_12_-chain at the lower rim (type II);
(3) Conformationally flexible thiacalixcrowns bearing OMe groups at the lower rim (type III).

**Scheme 1:**
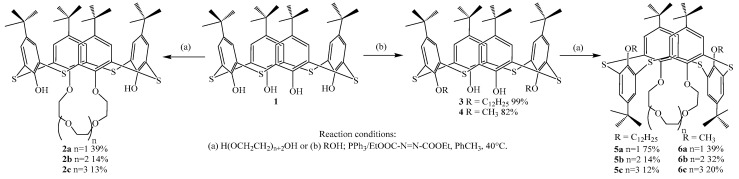
Synthesis of thiacalixcrown-ethers.

Crosslink of distal OH groups of thiacalixarene with oligoethylene glycols using triphenylphosphine and diethyl azodicarboxylate system (Mitsunobu reaction) or ditosylates through K_2_CO_3_-mediated nucleophilic substitution is a common macrocyclization route into thiacalixcrowns [23,24]. Former approach is preferable for dialkylated thiacalixarene precursors, because ether groups can be cleaved in alkaline medium at high temperature [25]. Type-I thiacalixcrowns **2a–c** were synthesized as previously reported from thiacalixarene **1** and tri-, tetra-, and pentaethylene glycol (Scheme 1) [23]. Given low yields of thiacalixcrowns **2b,c**, alkyl fragments were firstly attached to compound **1** to access type-II/III ligands. Dialkylated products **3** and **4** were synthesized as reported previously [26,27] and further reacted with the glycols (Scheme 1). Type-II/III crown-ethers **5b,c** and **6b,c** were isolated in low yields (12–32%), while yields of crown-4 **5a** and **6a** were 75% and 39%, respectively. These results agree with the reported increase in the yield of crown-4 derivatives as compared to larger crown-ethers, forming open-chain products and calixtubes [28,29].

**Figure 3:**
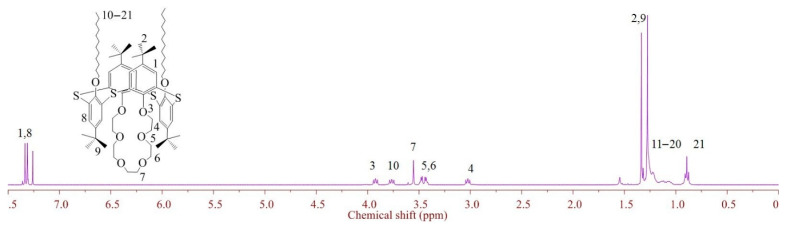
^1^H NMR spectrum of compound **5c** in CDCl_3_ at 293 K.

The physical characteristics of ligands **2a–c** coincide with the literature data [23] and indicate *cone* configuration due to the large chemical shift (CS) difference of aryl and *t*-Bu groups in ^1^H nuclear magnetic resonance (NMR) spectra. Intramolecular [1+1]-macrocyclization follows from matrix-assisted laser desorption–ionization (MALDI) mass peaks at *m*/*z* 857–946 (Figures S1–S6). CS of OH group in crown-4 **2a** is quite large as compared to that in crowns **2b,c** (8.46 ppm vs. 8.00–8.10 ppm) (Figure S1). Such features of dialkylated thiacalix[4]arenes were previously mentioned [30] and indicate that ligand **2a** exists as *pinched cone* (*PC*) stereoisomer, while ligands **2b,c** adopt *distorted cone* (*DC*) conformation (Figure S27). The structures of new ligands **5a–c** were assigned by ^1^H/^13^C NMR and infrared (IR) spectroscopy and MALDI mass spectrometry (Figures S7–S18). An example ^1^H NMR spectrum of ligand **5c** (Figure 3) shows a small CS difference of aryl and *t-*Bu protons (0.03–0.06 ppm) and upfield signals of ArOCH_2_ protons with respect to compound **2c** due to magnetic anisotropy of proximal inverted aromatic rings (4.01 vs. 4.74 ppm), which indicates *1,3-alternate* conformation. The crown-ether bridge peak order is another empirical criterion for elucidation of stereoisomeric form of thiacalixcrowns [24]. Namely, if the protons of oxyethylene unit next to aromatic ring are downfield with respect to other crown-ether protons, calixarene adopts the *cone* conformation, whereas these protons are most down- and upfield peaks in *1,3-alternate* conformation. In contrast to *cone* **2c** with peak order of 13–14–15–16–17 (Scheme S1), this order is 3–7–5–6–4 in *1,3-alternate* **5c** (Figure 3).

Monocrowns **6a–c** were originally identified as *1,3-alternate* stereoisomers due to high-symmetry ^1^H NMR spectra [13,24]. However, such assignment is in apparent contradiction with conformationally flexible thiacalix[4]arenes, bearing larger units at the lower rim [31,32]. NMR spectra reveal high-field shift of OMe group at 3.30–3.50 ppm, a large CS difference between the protons of benzene ring (0.14–0.19 ppm in crowns **6a,b** vs. 0.03 in crowns **5a,b**) and *t*-Bu groups (0.18 ppm in crown **6c** vs. 0.05 in crown **5c**), and violated crown-ether peak order in compounds **6b**,**c** (Figures S19–S21). These factors highlight that anisole rings decline from macrocycle rim and deshield aryl protons, whereas OMe groups approach proximal benzene rings of the macrocycle and shield them. Such behavior can be rationalized by a fast *1,3-alternate*–*partial cone* equilibrium. This suggestion is confirmed by a *1,3-alternate*-to-*partial cone* transformation upon crystallization of compound **6c** [24].

### 2.2. Complexation of Metal Ions at Liquid–Liquid Interface

**Figure 4:**
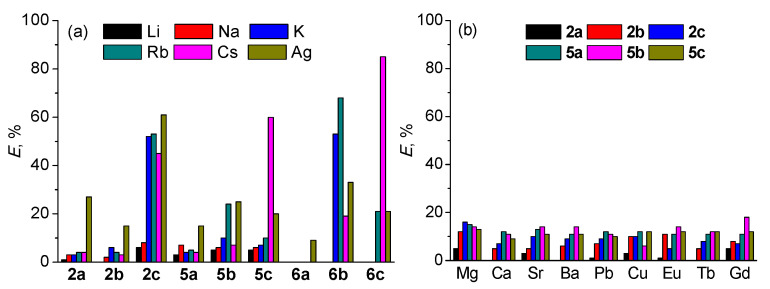
Degree of extraction (*Е*, %) of (**a**) alkali metal picrates (pH = 12) and AgPic (pH = 7) by compounds **2**, **5**, and **6** and (**b**) other metal picrates (pH = 5.8, tris buffer) by compounds **2** and **5**. *c*_M_ = 2 × 10^−4^ M, *c*_L_ = 4 × 10^−4^ M. The error is less than 2%.

Previously reported results of extraction of thiacalixcrowns **2b** and **6b**,**c** [13,33] cannot be compared due to different organic phase and component ratio. Thus, liquid-phase extraction of alkali metal and Ag picrates by type I–III ligands was carried out (Figure 4a). All the ligands extracted Ag^+^, with the degree of extraction from 9% in crown-4 **6a** to 61% in crown-6 **2c**. The main reason for Ag^+^ extractability is that S atoms are not much solvated in the aqueous phase. However, alkali metal ion extractability is governed by thiacalixcrown topology. Solvent-accessible crown-ether cavity in type-I *cone* thiacalixcrowns **2a–c** is influenced by the solvation of adjacent ionizable OH-groups along with H-bonding of aryl O atoms with OH groups. These factors lead to the higher energy of complexation involving desolvation of receptor fragment. Indeed, group IA metal ions were hardly extracted by compounds **2a,b**. Crown-6 macrocycle of compound **2c** is hindered by OH groups to a lower extent than smaller crown-ethers and displayed a group selectivity towards K^+^, Rb^+^, and Cs^+^, the extraction of Rb^+^ (33%) being highest. Such selectivity can be rationalized by lower free energy of transfer of Cs (25.73 kJ × mol^−1^), Rb (27.57 kJ × mol^−1^), and K (36.52 kJ × mol^−1^) picrates to CH_2_Cl_2_ compared to NaPic (61.84 kJ × mol^−1^) [34] and similar sizes of crown-ether and Rb^+^ ($d_{{\mathrm{Rb}}^{+}}$ = 3.1 Å [35]; *d*_crown_ = 3.2 Å [36]).

**Figure 5:**
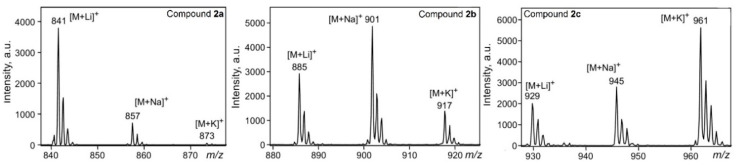
Matrix-assisted laser desorption–ionization (MALDI) mass spectra of the ligands **2a–c** mixed with metal nitrate salts.

A decisive role of solvation of type-I crown-ethers and metal ions in extraction was confirmed by the gas-phase analysis of crown-ethers **2a–c** with the mixture of metal nitrates (Figure 5). Under competitive conditions, the size complementarity of crown-ether and ion was revealed. Most intense mass peaks corresponded to the complexes of **2a**–Li^+^, **2b**–Na^+^, and **2c**–K^+^. All metal salts showed mass peaks corresponding to crown-ether complexes when added separately (Table S1), which excludes the factors beyond complexation, e.g., instability of molecular ions with other metals. Thus, Ag^+^ or alkali cation selectivity of compounds **2a–c** is governed by charge interactions with crown-ether and OH moieties in the gas phase and orbital interactions with sulfide bridges at the liquid–liquid interface.

In type-II/III ligands, crown-ether cavity is hindered from solvent molecules by *t*-Bu groups and benzene rings and impact of solvation on alkali metal extraction selectivity could be lower and resemble gas-phase ion selectivity. Extraction experiments confirm this suggestion and ionic radius–crown-ether size correlation was observed, with the highest degree of extraction of K^+^/Rb^+^ by crown-5 receptors **5b** and **6b** and Cs^+^ by crown-6 receptors **5c** and **6c**. The reported alkali metal ion extraction selectivity of crown-ethers **6b**,**c** at higher concentrations (10^−3^ *M*) [13] agrees with extraction data of this work and gas-phase complexation of *1,3-alternate* thiacalixcrowns **5a–c**, which were selective towards Na^+^, K^+^, and Cs^+^, respectively (Figure S22). A different size complementarity of metal ions with compounds **5a–c** and **2a–c** in the gas phase is presumably caused by different accessibility of crown-ether units and ion coordination by phenyl rings in former case and OH groups in latter case.

Unlike thiacalixcrowns-5(6), crown-4 ligands are poor extractants, which indicates the role of the factors other than solvent accessibility of crown-ether macrocycle. Quantum-chemical modeling of Li^+^ complexes of 12-crown-4 and compounds **2a**/**6a** (Table S2) revealed their different shapes. In contrast to Li^+^ complex of 12-crown-4, where the *d*_Li–O_ is ca. 2.0 Å and *d*_O–O_ are ca. 2.7 Å, the crown-ether in ligands **2a**/**6a** is distorted, with distances of 4.1–4.6 Å between distal O atoms. Such expansion of crown-ether increases Li–O bond lengths and results in less effective binding. Although OH group in ligand **2a** can coordinate cations, the complexation constant should be low due to entropic penalty.

Surprisingly, ligands **2** and **5** could extract all cations, which is in sharp contrast to the behavior of compounds **6b,c** and other *1,3-alternate* (thia)calix[4]crowns [13]. The scope of ions was extended to group-II metal ions, Pb^2+^, Cu^2+^, and lanthanide ions and 5 to 20% of these ions were extracted into CH_2_Cl_2_ (Figure 4b). With regards to ion extraction by type-I ligands **2a–c**, high pH at which phenolate anions could be involved in charge interactions with metal ions needs to be considered. Moreover, amphiphilic ligands **2a–c** and **5a–c** could display surface activity, which is discussed in Section 2.3.

### 2.3. Aggregation of Crown-Ether Ligands with and without Metal Ions

Exact mechanism of metal ion extraction is yet unclear, but assistance of complexation-induced micelle formation in the aqueous phase [37] and organic phase [38,39] to the metal ion transfer through the water–oil interface was claimed and recently reviewed [40]. A number of methods was employed to ensure the absence of calixcrown aggregates in organic solvent before establishing the role of aggregation in cation extraction. The dynamic light scattering (DLS) technique showed no spherical particles of crown-ethers **2**, **5**, and **6** in CHCl_3_ at *c* = 4 × 10^−4^ M. Formation of true solutions of type-II ligands **5** was supported by Fourier transform pulsed-gradient spin-echo (FT-PGSE) NMR experiments. Self-diffusion constants of amphiphilic calixarene **5a** and reference compound **7** [29] are similar in CDCl_3_ at higher concentrations and their aggregation numbers are close to 1 (Table 1).

Having excluded the aggregation of calixcrowns in individual solvent before extraction, particle size distribution (PSD) in CH_2_Cl_2_ and aqueous phases after their mixing was analyzed. Table 2 shows number-averaged PSD of crown-6 **2c**, **5c**, and **6c** after mixing in CH_2_Cl_2_–water system with and without Cs salts (PSD plots and correlation functions are given in Figures S23–S25). There is a different pH in blank aqueous phase (pH ≈ 7) and the aqueous phase with CsPic (pH ≈ 12); moreover, there is a possible effect of Pic^−^ on the aggregation of calixcrowns. Therefore, CsNO_3_ was also tested due to its neutral pH, which allows for a direct evaluation of the role of Cs^+^ on the PSD.

Blank experiments with Cs salts verified the absence of any aggregates after mixing CH_2_Cl_2_ with aqueous salt solutions. When thiacalixcrowns were dissolved in CH_2_Cl_2_, true solutions were no longer detected. After mixing with water, type-I ligands with OH-groups formed microparticles in CH_2_Cl_2_ resulting in the turbid organic phase. Lipophilic dodecyl derivative **5c** formed polydisperse nanoassociates in CH_2_Cl_2_ with the *R*_h_ = 142 nm and no aggregates in the aqueous phase. More hydrophilic methoxy derivative **6c** assembled into 300-nm aggregates in the aqueous phase, whereas only microparticles were detected in CH_2_Cl_2_. It can be hypothesized that mixed solvent alters hydrophilic–lipophilic balance of calixcrowns due to inclusion of solvent into macrocycle cavity and facilitates aggregation in the form of microemulsion. The density functional theory (DFT) study of solvation of crown-6 receptors confirms that macrocycle **5c** is most lipophilic, with the Δ*G* = 96.23 kJ × mol^−1^ between CHCl_3_ and water, whereas the energy gap corresponds to 46.02 and 20.92 kJ × mol^−1^ in ligands **2с** and **6с**. Thus, the results of DFT calculations rationalize the absence of aggregates of **2c**/**5c** in the aqueous phase due to low probability of crossing the water–oil boundary.

Two trends became visible on addition of Cs salts to the aqueous phase. Firstly, nanoassociates were detected in the organic phase after extraction, which suggests complexation-induced aggregation of calixcrowns after metal-ion transport through the oil/water interface. Secondly, and more interestingly, the aggregates of type-I/II amphiphiles **2c** and **5c** were also detected in the aqueous phase, whereas type-III compound **6c** did not form nanoassociates and no particles were detected in the aqueous phase after extraction of Cs salt. The particle size in the aqueous phase after CsNO_3_ extraction by both types of amphiphilic ligands was ca. 300 nm with a polydispersity index of ca. 0.4. The mean aggregate size in the aqueous phase decreased to 81 nm when CsPic was extracted by dodecyl derivative **5c**, which is presumably caused by higher lipophilicity of Pic^−^ as compared to ${\mathrm{NO}}_{3}^{–}$. In contrast, no aggregates of ionizable thiacalixcrown-6 **2c** with CsPic were detected in both phases at high pH due to phenolate ion formation, which disfavors the association of Cs^+^ complexes.

Analysis of PSD showed the connectivity of aggregation phenomena with metal ion extraction by amphiphilic calixcrowns and agrees with literature data on Am^3+^ and Cs^+^ selective calix[8]arenes [41] and Ag^+^-selective thiacalixtubes [42]. In spite of insufficient data set to establish the mechanism of picrate extraction, the results of DLS in the aqueous phase suggest nonspecific inclusion of metal picrates to calixcrown aggregates, which leads to their nonzero extractability. In contrast to type-I/II receptors, disappearance of nanoassociates of flexible methoxy derivative **6c** on addition of salt to the aqueous phase agrees with the proposed mechanism of Cs^+^ extraction by methoxy derivative of calixcrown counterpart “right at the interface” [43] and is presumably caused by the salting-out effect, which increases the critical aggregation concentration of compound **6c** in the aqueous phase.

The statements derived from the DLS study of type I–III crown-6 receptors after Cs^+^ extraction were also valid for other metal salts, such as NaPic and RbPic. In spite of low degree of Na^+^ and Rb^+^ extraction, nanoaggregates were detected in type-I/II conjugates in contrast to type-III methoxy derivative (Figure S26). Thus, evidence that nonzero metal ion extractability by type-I/II crown-ether receptors is related to surface activity in the aqueous phase was provided.

### 2.4. Langmuir Monolayer Formation

**Figure 6:**
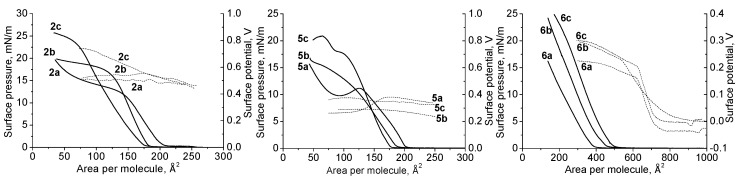
Surface pressure/surface potential (SPOT)–molecular area isotherms of compounds **2a–c**, **5a–c**, and **6a–c** at the air–water interface (*c* = 1 × 10^−4^ *M* (**2a–c**, **5a–c**) and 1 × 10^−5^ *M* (**6a–c**) in CHCl_3_).

Formation of true solutions of the thiacalixcrowns in CHCl_3_ indicated its feasibility as a spreading solvent to form Langmuir monolayers at the air–water interface. The importance of the absence of aggregates in spreading solution for true monolayer production was recently demonstrated for nitrothiacalix[4]arenes [44]. Structural optimization of the thiacalixcrowns using molecular mechanics (Figure S27) shows that type-I crown-ethers **2a–c** occupy the area of ca. 200 Å^2^ per molecule at parallel orientation of С_2v_ axes of molecules relative to water subphase, whereas *1,3-alternate* ligands occupy ca. 250 Å^2^ (dodecyloxy derivatives **5a–c**) and 125–163 Å^2^ (methoxy derivatives **6a–c**) at the same orientation (Table 3, parameter $A_{∥}$). Therefore, aliquots of 1 × 10^−4^ *M* solutions of crown-ether receptors in CHCl_3_ were spread over water subphase to give the starting molecular area larger than their parallel orientation and surface pressure/surface potential (SPOT)–molecular area isotherms were recorded upon monolayer compression (Figure 6).

The ligands formed rigid monolayers with an upward trend between the size of the crown-ether unit and the bend pressure of monolayer isotherms due to the increase in hydrophilicity and size of the crown-ether macrocycle (Figure 6). Compression moduli ($C_{s}^{–1}$) of the ligands are 25–72 mN/m, which corresponds to the liquid-expanded state of the monolayers [45]. UV/visible reflection–absorption spectra (UVRAS) of ligands **5a–c** and **6a–c** in monolayers are similar to their electronic absorption in CHCl_3_ (*λ*_max_ = 270–273 nm) (Figure S29a,b), whereas the absorbance maximum in spectra of compounds **2a–c** appears at 302–304 nm. To ensure that this absorbance maximum corresponds to the second band in solution spectra rather than a red-shifted band at 284 nm due to solvatochromism, the MeOH cosolvent was gradually added to 1 × 10^−4^ *M* solution of compound **2c** in CH_2_Cl_2_. As expected, no shift of the absorbance peaks at 281 and 303 nm was observed (Figure S29c).

Type-I/II ligands **2a–c** and **5a–c** are amphiphilic and crown-ether cavity should be immersed into water subphase upon spreading, with the perpendicular orientation of C_2v_ axes relative to the water surface, which is confirmed by the surface dipole moment of 0.7–2.0 D from SPOT measurements. The take-off area of compounds **5a–c** (177–201 Å^2^) is less than the calculated area of parallel-oriented molecules, whereas the take-off area of compounds **2b,c** (176–182 Å^2^) is similar to parallel orientation (Table 3). Crown-4 **2a** is characterized by larger take-off area (195 Å^2^) due to *PC* conformation of thiacalixarene, whose calculated area ($A_{⊥}$) is largest among type-I receptors (117 Å^2^ vs. 103–104 Å^2^). Unlike compounds **2** and **5**, the orientation of less lipophilic type-III ligands **6a–c** in monolayer is unclear a priori. These compounds did not form reproducible Langmuir monolayers at the spreading area of 300 Å^2^ per molecule. Therefore, their spreading area was increased up to 1000 Å^2^ to give true monolayers with the takeoff areas of 400–500 Å^2^ per molecule. These differences in mean molecular areas appear to be related to significant conformational changes in the molecules of compounds **6a–c** (previously mentioned in Section 2.1) upon transition from the organic phase to the air–water interface. Similar values were reported for non-amphiphilic terpyridine-thiacalix[4]arenes [46].

Upon monolayer compression, the SPOT values of ligands **2a–c** and **5b,c** increase slightly from ca. 450 mV and 100–200 mV, respectively, before collapsed state of monolayer, which shows dipole ordering at spreading areas and no further reorientation. The SPOT values of ligand **5a** gave a downward trend at *π* > 6 mN/m, indicative of onset of multilayer formation. In contrast, near-zero SPOT values of type-III compounds **6a–c** at the spreading areas suggest no aggregates/islands in monolayers. Moreover, critical molecular areas corresponding to a drastic rise of SPOT and structuring of monolayer in the form of islands (700–800 Å^2^) are much higher than take-off areas in *π*–*A* isotherms (400–500 Å^2^).

**Figure 7:**
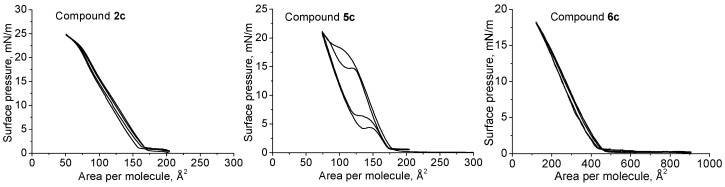
Monolayer compression–expansion cycles of compounds **2c**, **5c**, and **6c** at the air–water interface (*c* = 1 × 10^−4^ *M* (compounds **2c** and **5c**) and 1 × 10^−5^ *M* (compound **6c**) in CHCl_3_).

Compression–expansion cycles were carried out to assess molecular interactions in a monolayer (Figure 7). No hysteresis loop was recorded in type-I calixcrown **2c**, which indicates a weak interaction between calixarene molecules and reversibility of the monolayer. A slight loss in the take-off area after the first cycle may result from residual patches in film after expansion. Normalized area relaxation curves (Figure S28) demonstrated high stability of compound **2c** monolayer near collapse pressures and no loss of molecular area over time. This fact indicates weak interactions and is in good agreement with the abovementioned behavior of compound **2c**, whose molecules do not form 3D aggregates at high surface pressures. In contrast, monolayer of ligand **5c** is evidently stabilized by hydrophobic interactions between dodecyl chains and a hysteresis loop was observed. These interactions are strong enough to change the monolayer irreversibly, because there is a drop of collapse pressure after the second compression cycle (15 ± 1 mN/m). A decisive role of C_12_-chains of crown **5c** rather than stacking of aryl rings in stabilization of associates formed upon monolayer compression was confirmed by nearly identical compression–expansion isotherms of methoxy counterpart **6c**.

The monolayers of ligands **2**, **5**, and **6** were deposited onto quartz wafer using the Langmuir–Blodgett technique. The transfer ratio (TR) was close to unity (Table 4) and indicated complete transition of the monolayers from air–water interface to substrate. Morphological characterization of the monolayers by atomic force microscopy (AFM) revealed formation of quasi-continuous films, with the 100–200 nm associates that are 3 to 9 nm in height (as indicated by pseudocolor scale) (Figures S31–S35). Surface roughness *R*_a_, which could be regarded as effective thickness of such films, corresponds to 0.25 to 2.50 nm and exceeds that of bare quartz (0.20 nm, Figure S30), showing that there is one-molecule-thick film.

### 2.5. Metal Ion–Monolayer Interactions at the Air–Water Interface

The electrostatic repulsion of metal ions in the monolayer and its further destabilization make it convenient to analyze the change of *π*–*A*/Δ*V*–*A* isotherms in conformationally fixed calixarenes over salt water subphase as a criterion of ion–monolayer interactions [47]. Optical response could also be useful to evaluate cation–π interactions in type-II thiacalixcrowns and monitor changes of calixarene conformation and aggregation. Thus, surface pressure and SPOT isotherms along with UVRAS were recorded to analyze ion uptake at the air–water interface. Complexation of calixarenes **2a–c** and **5a–c** was studied by spreading the ligand from the organic solvent over the water subphase containing metal nitrates and subsequent monolayer compression; take-off areas of the ligands are given in Table 5.

**Figure 8:**
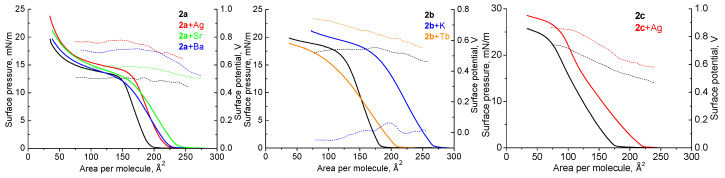
Surface pressure/SPOT–molecular area isotherms of monolayers of thiacalixcrowns **2a–c** on water or 1 × 10^−2^ *M* salt water subphase. Concentration in CHCl_3_ is 1 × 10^−4^ *M*.

Langmuir monolayers of type-I crown-4 **2a** marginally change the take-off area of 195 Å^2^ in the presence of most metal ions in water subphase, which agrees with the predicted distortion of crown-ether cavity (Table S2). However, the monolayer expanded significantly in the presence of Ba(NO_3_)_2_ and Sr(NO_3_)_2_ in the water subphase, which gave the values of 226 and 241 Å^2^/molecule (Figure 8). In addition, SPOT values of the monolayer of compound **2a** increased in the presence of Sr^2+^/Ba^2+^, 580/710 mV vs. 508 mV over deionized water (Figure 7) due to the decrease in the negative contribution of the double-layer potential of ionized monolayers. Given the small size of the crown-4 cavity, the binding of these ions could be rationalized by exocyclic coordination with OH groups and a crown-ether fragment. The distances between oxygen atoms of the crown-ether ring and OH groups are ca. 3.0 Å in compound **2a** (Table S2), which fit well Ba^2+^ and Sr^2+^ ions (*d*_ion_ = 2.36–2.70 Å [35]).

The monolayer of crown-5 **2b** expanded in the following order: K^+^ > Rb^+^ > Cs^+^ (Figure 8), while minor changes were observed with alkaline-earth metal ions (Table 5). The Δ*V*_max_ value of compound **2b** with K^+^, Rb^+^, and Cs^+^ decreased from 517 to −50, −8, and 155 mV, which can be related to the conformational change of the crown-ether ring. The recovery of alkali metal uptake by this ligand could be associated with a near-neutral pH, at which the crown-ether macrocycle is less solvated than at high pH values used for picrate extraction (pH = 12) due to OH groups, in addition to increased steric hindrance from solvent molecules due to lateral compression. More compressible monolayers were recorded over the Pb^2+^/Tb^3+^ salt water subphase and SPOT increased to ca. 740 mV. A decrease in the $C_{s}^{–1}$ values of the latter monolayers can be caused by ion binding by OH groups through deprotonation. The fact that all metal ions, which expanded the monolayer of compound **2b**, are larger than Na^+^ (except for Li^+^), which was effectively encapsulated by the crown-5 cavity of thiacalixarene in the gas phase (Figure 5), again suggests exocyclic coordination of metal ions with crown-ether and OH moieties.

The largest monolayer expansion of crown-6 **2c** was recorded over water subphase containing AgNO_3_ (Figure 8), the collapse pressure of monolayer increased from 22 to 24 mN/m, and Δ*V*_max_ value also increased from 740 to 860 mV, whereas K^+^, Rb^+^, and Cs^+^ expanded the monolayer of compound **2c** to a lower extent. Thus, there is an apparent Ag^+^ binding of compound **2c** at the air–water interface. UVRAS spectra of the monolayers over different subphases were nearly identical, which indicates retention of the conformation of calixarene macrocycle (Figure S36a).

**Figure 9:**
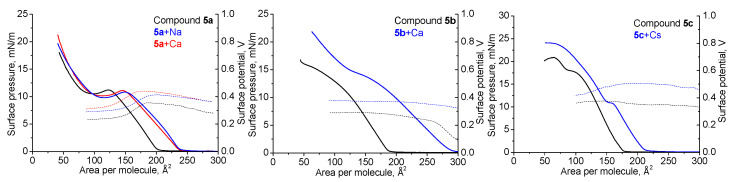
Surface pressure/SPOT–molecular area isotherms of monolayers of thiacalixcrowns **5a–c** on water or 0.01 *M* aqueous salt solution subphase. The concentration in CHCl_3_ is 1 × 10^−4^ *M*.

Unlike type-I ligands, cation-π interactions could govern metal ion uptake of *1,3-alternate* thiacalixcrowns due to proximity of aryl rings to crown-ether macrocycle, which is particularly important in the case of alkaline-earth metal ions [48]. Na^+^ and Ca^2+^ salts shifted the pressure–area isotherm to larger areas and increased surface potential of type-II thiacalixcrown **5a** (Figure 9). Similarly, the largest monolayer expansion of crown-5 **5b** was recorded in the presence of Ca^2+^, and SPOT increased to 376 mV. A red shift of the absorption band from 273 to 290 nm in UVRAS spectra at high surface pressures indicates either cation–π interactions of Ca^2+^ with benzene rings of thiacalixcrown **5b** (previously indicated in metal complexes of *1,3-alternate* calixcrowns [9]) or aggregation of molecules (there is a shoulder band in UV/visible spectra of the ligands at 290 nm), whereas UVRAS spectra of compounds **5a** and **5c** did not change over salt water subphase (Figure S36b–d). Ca^2+^ binding by crown-ethers in type-II ligands **5a,b** reveals the contribution of the pre-organization of calixarene in the *1,3-alternate* configuration to favor cation–π interactions and Ca^2+^–crown-ether size complementarity. When there is CsNO_3_ in the water subphase, the monolayer of crown-6 **5c** is expanded to the take-off area of 168 Å^2^, whereas Δ*V*_max_ increases to 509 mV. The kink at *π* = 10 mN/m is accompanied by the onset of SPOT decrease, which suggests the onset of multilayer formation.

Type-III ligands **6a–c** displayed opposite trends in surface pressure–area isotherms (monolayer compression and expansion) over the salt water subphase, which can result from their flexibility or large molecular areas in the monolayer (Figure S37); thus, no clear statements on the ion uptake can be made.

The expansion of the monolayers of type-I/II crown-ethers on addition of salts to the water subphase allows to suggest that monolayer is significantly destabilized due to electrostatic repulsion between metal ions. To confirm monolayer destabilization and establish ion selectivity by thiacalixcrowns, the change of the Gibbs energy for monolayer-expanding metal ions was calculated:

$$\Delta G=N_{A}\left(\overset{\pi_{0}}{\underset{0}{\int }}A_{\mathrm{salt}}d\pi -\overset{\pi_{0}}{\underset{0}{\int }}A_{\mathrm{water}}d\pi \right),$$

where *π*_0_ is the upper limit of surface pressure and *A*_salt_ and *A*_water_ are the areas per molecule on the aqueous salt solution or pure water subphases, and *N*_A_ is the Avogadro’s number [49]. The upper limit of surface pressure was taken to represent the monolayer before collapse and is specified in Table 6. In agreement with the largest monolayer expansion, the largest Δ*G* values were determined in the case of thiacalixcrowns **2a–c** and **5a–c** over the corresponding metal salt solutions.

**Figure 10:**
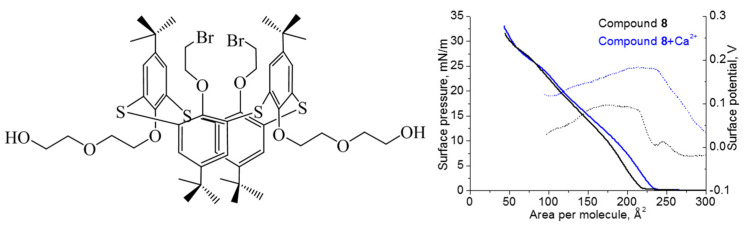
The surface pressure/SPOT–area isotherms of monolayers of compound **8** on water or 1 × 10^−2^ *M* Ca(NO_3_)_2_ subphase and the proposed orientation of compound **8** in the monolayer. *c* = 1 × 10^−4^ *M* in CHCl_3_.

Monolayer expansion of type-II receptors **5a–c** in the presence of Ca(NO_3_)_2_ in the water subphase could suggest sole responsibility of *1,3-alternate* topology for Ca^2+^ selectivity. To verify this hypothesis, a linear analog of thiacalixcrowns **5a–c**, compound **8** with two oxyethylene arms [17], was spread on the water subphase and its monolayer characteristics were measured (Figure 10). In analogy to the thiacalixcrowns, it was characterized by a take-off area of 217 ± 7 Å^2^ and a liquid-expanded state of the monolayer ($C_{s}^{–1}$ = 37 mN/m), whereas near-zero SPOT could be caused by parallel arrangement of oxyethylene chains at the air–water interface. On addition of Ca(NO_3_)_2_ to the water subphase, the monolayer slightly expanded to 234 ± 7 Å^2^ (Δ*G* = 1.08 kJ × mol^−1^ at *π*_0_ = 15 mN/m) and SPOT increased from 90 to 182 mV, whereas the UVRAS spectra did not change (*λ*_max_ = 271 nm, Figure S38). These experiments demonstrate that the assembly of oxyethylene units into crown-4–6 macrocycle on *1,3-alternate* topology are crucial for effective Ca^2+^ uptake at the air–water interface.

**Figure 11:**
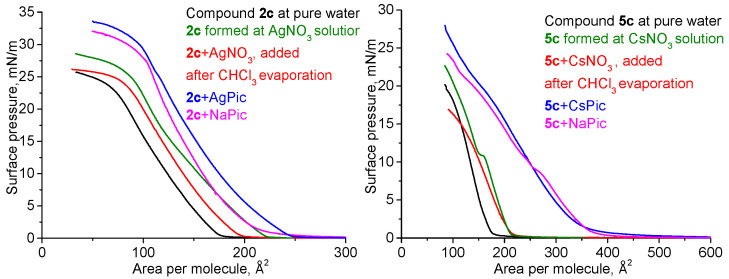
The surface pressure/SPOT–molecular area isotherms of monolayers of thiacalixcrowns **2c** and **5c** on water or 1 × 10^−2^ *M* aqueous salt solution subphase. The concentration in CHCl_3_ is 1 × 10^−4^ *M*.

Previous experiments with crown-ether hemicyanine dyes confirmed that effective “oxygen crown-ether–metal ion” interactions occur only at initial stage of monolayer formation before CHCl_3_ evaporation [50,51,52], and this interaction can alter the molecular arrangement in the monolayer. According to this model, the influence of cation on monolayer formation correlates with ion–receptor complementarity. To reveal the effect of residual CHCl_3_ solvent on cation binding by crown-ether fragment in the ligands at the air–water interface, the ligand **5c** was spread over pure water subphase and, as CHCl_3_ evaporation was complete, the aliquot of CsNO_3_ was added to the water subphase under the monolayer. The maximum surface pressure for so-formed monolayer decreases as compared to one formed on CsNO_3_ solution, indicating that the associates formed upon monolayer compression are destabilized (Figure 11). The kink at *π* = 10 mN/m also disappears; however, the take-off area remains the same, which allowed us to state that Cs^+^–thiacalixcrown interactions could occur without CHCl_3_ and the change of take-off area is a reliable criterion of ion–ligand interactions for the thiacalixcrowns under study. On the contrary, crown-ether cavity is more accessible for solvent molecules in type-I receptors **2a–c** and the effect of residual CHCl_3_ solvent on ion–monolayer interactions could be remarkable. A comparison of the *π*–*A* isotherm of the monolayer of compound **2c** over the pure water subphase with those for the monolayers formed over the AgNO_3_ aqua solution subphase and ones under which AgNO_3_ was added after CHCl_3_ evaporation confirmed the role of organic solvent in ion interactions and showed that the monolayer expansion is lower when the salt is added after the evaporation of CHCl_3_.

It could be suggested that picrate salts facilitate cation uptake by crown-ethers as compared to nitrate salts (by decreasing the energy of complexation and energy of phase transfer). A difference between the *π*–*A* isotherms of the monolayers of compound **2c** on AgPic and NaPic solutions (Figure 11) shows that Ag^+^ vs. Na^+^ selectivity is preserved in the case of picrate salts. Moreover, larger shifts of the take-off areas correlated with the larger destabilization of compound **2c** monolayer over picrate salt solution as compared to that over nitrate salt solution (Δ*G* = 6.56 kJ × mol^−1^ with AgPic vs. Δ*G* = 3.76 kJ × mol^−1^ with AgNO_3_). In contrast, the *π*–*A* isotherms of the monolayer of type-II compound **5c** recorded over NaPic or CsPic solutions were identical except for the kink at *π* ≈ 10 mN/m in case of NaPic. Such coincidence of the isotherms could be interpreted as a trend of hydrophobic Pic^−^ to form mixed monolayers with surfactants at the air–water interface through interactions between aromatic rings [53], which hampers analysis of cation binding from monolayer isotherm data.

Ion binding selectivity by thiacalixcrowns is summarized in Table 7 and again emphasizes the effect of topology of the ligands and environment. Gas-phase ion selectivity is governed by charge interactions and size fit of crown-ether unit with metal ions, which results in the most abundant peaks of alkali metal complexes of calixcrown-ethers, whereas liquid-phase extraction conditions favor Ag^+^ binding due to solvated crown-ether cavity, and alkali metal ion selectivity could arise only when the crown-ether is hindered from solvent molecules (type-II receptors **5b,c**). The origin of a different ion selectivity of the crown-ether ligands at the air–water interface is considered in Section 2.6.

### 2.6. Mechanism of Metal Ion Binding by Thiacalixcrowns at Different Interfaces

**Figure 12:**
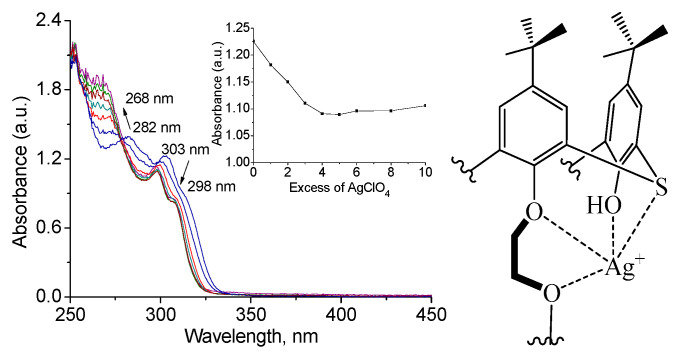
Evolution of UV/visible absorption spectra of compound **2c** (*c* = 1 × 10^−4^ *M* in CH_2_Cl_2_) on the addition of 1–10 eq. AgClO_4_ and proposed binding mode of compound **2c** with Ag^+^ ions in solution.

The contribution of solvent seems to result in identical ion selectivity of the crown-ether ligands **2** and **5** at CH_2_Cl_2_–water and air–water interfaces. However, a comparison of ion binding selectivity under these conditions (Table 7) reveals a number of differences. Firstly, there is no change of the *π*–*A* isotherms of type-II thiacalixcrowns over AgNO_3_ water subphase. To reveal a binding motif of Ag^+^ ions and verify the role of S atoms in extraction of crown-ether ligands that was postulated in Section 2.2, complexation-induced shifts of NMR resonances of compound **6c** were analyzed (Table 8). On addition of 1 eq. AgClO_4_ to compound **6c** in CDCl_3_ (Figure S39), the signals of crown-ether are deshielded by 0.20–0.27 ppm, which indicates its coordination. Proximal Ph units could be involved in Ag^+^ complexation through ion–π interactions or orbital interactions with S bridges, as evidenced by their downfield shift by 0.10 ppm. NMR spectra were recorded 1 h after preparation of solution and did not change within next 24 h, as well as upon the addition of larger amounts of AgClO_4_. Therefore, the absence of monolayer expansion of type-II thiacalixcrowns can be rationalized by steric hindrance of S atoms at air–water interface. Consequently, ion binding selectivity under these conditions is governed by the size fit of crown-ether cavity and metal ion in analogy to the gas-phase interactions (compound **5a**–Na^+^ and compound **5c**–Cs^+^, Table 8). In contrast to *1,3-alternate* stereoisomers, a positive cooperativity between S and O atoms can be achieved in *cone* thiacalixcrowns **2a–c** upon Ag^+^ binding. There are downfield shifted signals of benzene rings (ca. 0.10 ppm) and upfield shifted signal of the ArOCH_2_ unit in ^1^H NMR spectrum of compound **2c** (Table 8, Figure S39), which is indicative of O_3_S binding mode of Ag^+^. Blue-shifted π–π* absorption bands at 282 and 303 nm, along with a hyperchromic effect in the former case in UV/visible spectra of compound **2c** with 1–10 eq. AgClO_4_ (Figure 12), further support the involvement of OH groups and S atoms in Ag^+^ binding. A decrease in the absorbance of crown-ether **2c** at 303 nm up to 4 equiv. of AgClO_4_ suggests that one molecule of thiacalixcrown receptor binds several Ag^+^ ions and is evidenced by the MALDI mass peaks corresponding to [M-H+2Ag]^+^ and [M-2H+3Ag]^+^ (Table S1). Notably, the signal of CH_2_-1 shifts upfield, which can be caused by the cleavage of H-bonds with OH group. Given a downfield shift of CH_2_-2,3, these results suggest exocyclic O_3_S coordination of Ag^+^ with phenolic O atom and two O atoms in crown-ether (Figure 12).

In spite of the ion selectivity of compound **2c** towards Ag^+^ both at air–water and CH_2_Cl_2_–water interfaces (Table 6), the involvement of S atoms in Ag^+^ binding at the air–water interface could not be deduced, because they seem not to be immersed into the water subphase due to their hydrophobicity, and there is an air–water boundary between S bridges and Ag^+^ ions. To estimate the role of S atoms in compound **2c** in Ag^+^ binding in Langmuir monolayers, monolayer characteristics of model compound **9** in *cone* topology, with bromopropyl fragments instead of crown-ether macrocycle, over pure water and AgNO_3_ water subphases were compared (Figure S40). Nearly identical *π*–*A* isotherms of monolayers in both cases indicate that OH groups without crown-ether fragment cannot effectively bind Ag^+^ ions at the air–water interface. Exocyclic coordination of Ag^+^ with compound **2c** suggests that crown-ether cavity in type-I ligands could not accommodate metal ions effectively due to solvation. A synergism between crown-ether and OH moieties could, thus, be proposed to interpret binding selectivity at air–water interface towards metal ions, of which the radius is larger than crown-ether cavity (compound **2a**—Sr^2+^ and compound **2b**—K^+^, Table 6).

Ion selectivity of crown-ether conjugates in compounds **2a** and **5b** towards Sr^2+^ and Ca^2+^ (Table 6) is particularly interesting in spite of the evidence of alkali metal binding at the air–water interface. This difference poses two questions: (1) why there was no extraction selectivity towards Ca^2+^ by ligand **5b** and Sr^2+^ by ligand **2a**; (2) why should there be selectivity towards mentioned metal ions vs. alkali metal ions at the air–water interface. The first question can be addressed by recalling a contribution of ion transfer from water to CH_2_Cl_2_ into extraction, because multiple-charged Ca^2+^ and Sr^2+^ ions require higher energy for transfer than single-charged alkali-metal and Ag^+^ ions. The second question was partially addressed in Section 2.5 regarding exocyclic coordination of alkaline-earth metal ions by type-I calixcrown **2a** and cation–π interactions of alkaline-earth metal ions with crown-ethers **5a,b** under lateral confinement. Due to the fact that the crown-ether unit is solvated in type-I receptors and the size fit of the crown-ether cavity and alkali metal ion is no longer decisive, a double-charged Sr^2+^ could stabilize exocyclic coordination mode in compound **2a** better than single-charged alkali metal ions. In analogy, solvation of crown-ether cavity in type-II receptors could decrease the contribution of the size fit in compound **5b**, making the cation–π interactions more important in ion selectivity, which are again stronger in the case of Ca^2+^ ions.

**Figure 13:**
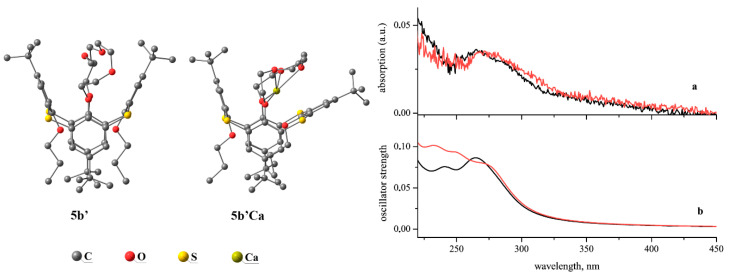
Optimized structures of model ligand **5b’** and complex **5b’**–Ca (left) and (right) (**a**) experimental UVRAS spectra of ligand **5b** over pure water subphase and over Ca(NO_3_)_2_ solution and (**b**) calculated UV spectra of ligand **5b’** and complex **5b’**–Ca (**5b**, **5b’**—black; **5b**+Ca(NO_3_)_2_, **5b’**–Ca—red).

To estimate the possibility of **5b**–Ca complex formation and its influence on UV/visible spectra, quantum chemical computations were carried out. To simplify geometry optimizations and calculations of vertical shifts at DFT, the structures of model compound **5b’** with a shorter propyl chain on the lower rim and corresponding **5b’**–Ca complex were considered. Crown-ether macrocycle of the complex is predicted to be more symmetrical than that of ligand and the crown-ether–Ca fragment is bent to the aryl ring that is declined from the C_2v_ axis of the molecule. This is accompanied by the formation of short contacts between Ca and O/ipso-C atoms of the neighboring aryl ring (2.64 and 2.65 Å, respectively) (Figure 13). Similar conformational changes of crown-ether unit in *1,3-alternate* calixcrowns upon complexation were evidenced in single crystals [54].

Theoretical UV/visible spectrum of **5b’**–Ca complex shows a ca. 10 nm red shift of the low-energy absorption band compared to the one of **5b’** (Figure 13b), in good agreement with the experimental data (Figure 13a). The analysis of the frontier molecular orbitals shows that the corresponding band in the spectrum of the ligand is caused by the intraligand charge transfer, whereas in the case of **5b’**–Ca, this band contributes to the ligand-to-metal charge transfer (Figure S41).

## 3. Materials and Methods

Organic solvents were purified by known procedures [55]; the reagents were used as received. Water was deionized on an Adrona Crystal purification system up to the conductivity of 0.055 µS/cm. Known procedures were employed to synthesize *p*-*tert*-butylthiacalix[4]arene **1** [56], dialkylated thiacalixarenes **3** [26] and **4** [27], and thiacalixcrowns **2a–c** [24] and **6a–c** [13,25].

All NMR experiments were performed with Bruker AVANCE-400/500 MHz (400.05/500.13 MHz for ^1^H NMR and 100.6/125.8 MHz for ^13^C NMR) spectrometers equipped with a Bruker multinuclear z-gradient inverse probe head capable of producing gradients of 50 G·cm^−1^. The NMR spectra were recorded at *T* = 295 and 303 K. Chemical shifts are reported in the *δ* (ppm) scale relative to the ^1^H (7.26 ppm) and ^13^C (77.2 ppm) signals of CDCl_3_ and coupling constants are denoted in Hz. The FT-PGSE experiments were performed by a bipolar pulse pair–stimulated echo-longitudinal eddy current delay (BPP-STE-LED) sequence [57] and repeated at least three times. All separated peaks were analyzed and the average values for the species were presented. The temperature was maintained at 303 K with a 640 L/h airflow rate to avoid heat fluctuations due to sample heating during the magnetic field pulse gradients. After Fourier transformation and baseline correction, the diffusion dimension was processed with the TopSpin3.2 program. Self-diffusion constants *D*_s_ were calculated by exponential fitting of the data from individual columns of the pseudo 2D matrix. Single components have been assumed for the fitting routine. Standard deviations of self-diffusion constants were less than 5%. Hydrodynamic radius *R*_h_ was calculated by the Einstein–Stokes equation:

$$R_{h}=\frac{k_{B}T}{6\pi \eta D_{s}},$$

where *k*_B_ is Boltzmann constant and *η* is dynamic viscosity of solvent (5.05 × 10^−4^ Pa s for CDCl_3_).

MALDI mass spectra were recorded on an UltraFlex III TOF/TOF mass spectrometer (Bruker Daltonik GmbH, Bremen, Germany) in a linear mode using a Nd:YAG laser, *λ* = 266 nm. The sample solutions were applied to the MTP AnchorChip^TM^ metallic target by the dried drop method. To evaluate gas-phase interactions with metal ions, an aliquot of 10 μL of a solution of thiacalix[4]arene-crown-ethers **2a–c** and **5a–c** (CHCl_3_, 1 mg/mL) was mixed with 10 μL of an aqueous solution of all metal nitrates under study at a concentration of 10 mg/mL. A total of 0.5 μL of the matrix solution and 0.5 μL of the sample were successively applied to the target and evaporated. *p*-Nitroaniline (10 mg/mL in CH_3_CN) was used as the matrix. Positively charged ions were recorded. The data were obtained using the Flex Control program and processed using the FlexAnalysis 3.0 program.

IR spectra were recorded in KBr on a Bruker Vector 22 spectrometer at *λ* = 400–4000 cm^−1^.

### Synthesis of Compounds ***5a*–*c***

Compound **3** (1 eq.), triphenylphosphine (6 eq.), and glycol (5 eq.) were suspended in 30 mL toluene and diethyl azodicarboxylate (6 eq.) was added dropwise at 0 °C. The reaction mixture was stirred 8 h, at 20 °C, and then for 8 h, at 40 °C. The solvent was removed under reduced pressure and methanol (50 mL) was added to the residue; the precipitate was centrifuged, filtered, and purified by column chromatography (hexane:ethylacetate = 4:1).

Compound **5a**. Yield 1.22 g (75%). *T*_m_ = 202 °C. *R*_f_ (*n*-C_6_H_14_:EtOAc = 6:1) 0.54. ^1^H NMR (CDCl_3_): 7.38 (s, 4H, H_12_), 7.32 (s, 4H, H_3_), 4.01 (m, 4H, H_7_), 3.78 (t, 4H, *J* 8.4, H_16_), 3.51 (m, 4H, H_8_), 2.51 (s, 4H, H_9_), 1.32 (s, 18H, H_6_), 1.29 (s, 18H, H_15_), 1.36–1.20 (m, 40H, H_17–26_), 0.90 (t, 6H, *J* 6.8, H_27_). ^13^C NMR (CDCl_3_): 158.1 (C_1i_), 156.4 (C_10i_), 146.1 (C_4i_), 145.9 (C_13i_), 128.9 (C_3_), 128.4 (C_2i_), 128.1 (C_11i_), 127.1 (C_12_), 71.3 (C_7_), 70.2 (C_16_), 68.8 (C_8_), 68.5 (C_9_), 34.5 (C_5i_), 34.4 (C_14i_), 32.1 (C_17_), 31.6 (C_15_), 31.5 (C_6_), 30.3 (C_18_), 29.8 (C_19–22_), 29.5 (C_23_), 28.5 (C_24_), 26.0 (C_25_), 22.9 (C_26_), 14.3 (C_27_). *m/z* (MALDI) (%) 1194.2 (100) [M+Na]^+^. Anal. calcd. for C_70_H_106_O_6_S_4_: % C 71.75, H 9.12; found: C 71.62; H 9.15. IR (KBr, $\tilde{\nu }$/cm^−1^) 2924 (C–H), 1436 (C_Ar_–C_Ar_), 1264 (C_Ar_–O–C_Alk_), 1086 (C_Alk_–O–C_Alk_). UV (CHCl_3_, 10^−5^ *M* (*ε*, *M*^−1^ cm^−1^)) 268 nm (35126).

Compound **5b**. Yield 0.24 g (14%). *T*_m_ = 161 °C. *R*_f_ (*n*-C_6_H_14_:EtOAc = 6:1) 0.30. ^1^H NMR (CDCl_3_): 7.34 (s, 4H, H_13_), 7.29 (s, 4H, H_3_), 3.91 (m, 4H, H_7_), 3.75 (t, 4H, *J* 8.2, H_17_), 3.60 (m, 4H, H_8_), 3.38 (m, 4H, H_9_), 2.99 (m, 4H, H_8_), 1.36 (s, 18H, H_6_), 1.28 (s, 18H, H_16_), 1.33–1.20 (m, 40H, H_18–27_), 0.90 (t, 6H, *J* 6.0, H_28_). ^13^C NMR (CDCl_3_): 156.5 (C_1i_), 156.4 (C_11i_), 146.1 (C_4i_), 145.9 (C_14i_), 128.1 (C_2i_), 127.8 (C_12i_), 126.9 (C_3_), 126.2 (C_13_), 73.7 (C_7_), 71.5 (C_17_), 70.4 (C_8_), 68.5 (C_9_), 65.5 (C_10_), 34.5 (C_5i_), 34.4 (C_15i_), 32.1 (C_18_), 31.7 (C_16_), 31.5 (C_6_), 30.2 (C_19_), 29.9 (C_20_), 29.8 (C_21–23_), 29.5 (C_24_), 28.8 (C_25_), 26.0 (C_26_), 22.8 (C_27_), 14.3 (C_28_). *m/z* (MALDI) (%) 1237.7 (100) [M+Na]^+^. Anal. calcd. for C_72_H_110_O_7_S_4_: % C 71.12, H 9.12; found: C 71.25, H 9.10. IR (KBr, $\tilde{\nu }$/cm^−1^) 2925 (C–H), 1442 (C_Ar_–C_Ar_), 1264 (C_Ar_–O–C_Alk_), 1089 (C_Alk_–O–C_Alk_). UV (CHCl_3_, 10^−5^ *M* (*ε*, *M*^−1^ cm^−1^)) 267 nm (29669).

Compound **5c**. Yield 0.22 g (12%). *T*_m_ = 211 °C. *R*_f_ (*n*-C_6_H_14_:EtOAc = 6:1) 0.21. ^1^H NMR (CDCl_3_): 7.34 (s, 4H, H_14_), 7.31 (s, 4H, H_3_), 3.92 (m, 4H, H_7_), 3.76 (t, 4H, *J* 8.2, H_18_), 3.55 (s, 4H, H_11_), 3.47 (m, 4H, H_9_), 3.44 (m, 4H, H_10_), 3.02 (m, 4H, H_8_), 1.33 (s, 18H, H_6_), 1.28 (s, 18H, H_17_), 1.29–1.03 (m, 40H, H_19–28_), 0.89 (t, 6H, *J* 6.8, H_29_). ^13^C NMR (CDCl_3_): 156.8 (C_1i_), 156.7 (C_12i_), 146.2 (C_4i_), 145.9 (C_15i_), 128.3 (C_2i_), 127.8 (C_13i_), 127.5 (C_3_), 126.7 (C_14_), 71.6 (C_7_), 71.4 (C_18_), 70.8 (C_8_), 69.6 (C_9_), 68.6 (C_10_), 67.2 (C_11_), 34.5 (C_5i_), 34.4 (C_16i_), 32.1 (C_19_), 31.6 (C_17_), 31.5 (C_6_), 30.2 (C_20_), 29.9 (C_21_), 29.8 (C_22–24_), 29.5 (C_25_), 28.7 (C_26_), 25.9 (C_27_), 22.9 (C_28_), 14.3 (C_29_). *m/z* (MALDI) (%) 1300.2 (100) [M+Na]^+^. Anal. calcd. for C_74_H_114_O_8_S_4_: % C 70.54, H 9.12; found: C 70.78, H 9.14. IR (KBr, $\tilde{\nu }$/cm^−1^) 2924 (C–H), 1444 (C_Ar_–C_Ar_), 1268 (C_Ar_–O–C_Alk_), 1091 (C_Alk_–O–C_Alk_). UV (CHCl_3_, 10^−5^ *M* (*ε*, *M*^−1^ cm^−1^)) 268 nm (38642).

Extraction of metal picrates from water into CH_2_Cl_2_ followed a typical procedure and was conducted in triplicates to ensure reproducibility. Picric acid (HPic), alkali metal hydroxides, and metal (Ca, Ba, Mg, Sr, Ag, Pb, Eu, Tb, Gd) nitrates were dissolved in H_2_O to the final concentration [HPic]_0_ = 2 × 10^−4^ *M* and [MOH(MNO_3_)]_0_ = 10^−2^ *M*. Tris buffer was added to metal nitrates (except for AgNO_3_) up to pH = 5.8 to avoid hydrolysis. Metal picrates were prepared by mixing aqueous solutions of HPic and metal hydroxides/nitrates in a 1:1 volume ratio. A total of 4 mL of ligands **2**, **5**, and **6** in CH_2_Cl_2_ (*c* = 4 × 10^−4^ *M*) was added to 4 mL of aqueous metal picrates. The biphase system was stirred for 30 min at room temperature and, then, maintained for 15 min for phase separation. The UV/Vis absorption spectra of the aqueous phase were recorded using an AvaSpec-2048 fiber optic spectrophotometer (Avantes, Apeldoorn, the Netherlands) in the wavelength range of 200–500 nm with a resolution of 1 nm before and after extraction and maximum absorbances (*А*_0_ and *А*_i_, respectively) were recorded at *λ*_max_= 355 nm. The extraction percentage (%*Е*) was calculated as follows:

$$\%E=\frac{A_{0}-A_{i}}{A_{0}}\times 100$$

DLS measurements were performed on a Zetasizer Nano particle size analyzer (Malvern Panalytical, Malvern, UK) in PCS1115 glass cuvettes thermostated at 25 °C. Three independent experiments were recorded for each sample; data were processed by Malvern DTS program.

The Langmuir method was used for monolayer formation by compounds **2**, **5**, and **6**. The UV/Vis absorption spectra of the CHCl_3_ solutions of thiacalixcrowns **2**, **5**, and **6** were recorded on an AvaSpec-2048 spectrophotometer to control their concentration before spreading onto the water subphase; the spectrophotometer was equipped with a reflectometric probe at a distance of ca. 1 mm from the water subphase and perpendicular to the water subphase to record UVRAS spectra of the monolayers using the method from [58]. Monolayers of the ligands **2**, **5**, and **6** were formed from CHCl_3_ solutions (1 × 10^−5^–1 × 10^−4^ *M*) spread onto the water subphase with a microsyringe at the air–water interface on a KSV NIMA Teflon trough (Biolin Scientific, Gothenburg, Sweden) equipped with a Pt Wilhelmy plate and two polyacetal barriers. SPOT was monitored (with an accuracy of ±2 mV) using KSV SPOT1 instrument (Biolin Scientific, Sweden) with a vibrating plate electrode at the distance of ca. 1 mm from the water subphase and a stainless steel counter electrode immersed in water. The surface dipole moment of molecules $\mu_{⊥}$ and compressibility modulus $C_{s}^{–1}$ were calculated as follows:

$$\mu_{⊥}=\frac{A\Delta V}{12\pi }\;;\;C_{s}^{–1}=-A{\left(\frac{\partial \pi }{\partial A}\right)}_{T},$$

where *A* is the molecular area, Δ*V* is surface potential at *A*, and *π* is surface pressure [45].

The following monolayer production conditions were employed: the time of spreading solvent evaporation was 15 min, monolayer compression rate was 5 cm^2^/min, and *t* = 25 °C. The KSV Nima/Attension 2.2 and Origin programs were used for processing surface pressure/SPOT–molecular area data. Monolayers were vertically deposited onto quartz (Flyuorit, St.-Petersburg, Russia), which was degreased with EtOH and washed with water before use. A 10^−2^ *M* solution of metal salts in water subphase was used for complexation study.

AFM measurements were carried out on a MultiMode V scanning probe microscope (Veeco, United States) in tapping mode using RTESP rectangular cantilevers (Veeco) equipped with silicone probes. Resonance frequency of these cantilevers falls within the range of 250–350 kHz and the probe curvature radius is 10–13 nm. Microscopy images were recorded at the resolution of 512 × 512 points per frame at the scanning rate of 1 Hz; external distortions with a frequency down to 0.5 Hz were eliminated using an SG0508 anti-vibration system. Results of AFM scanning were processed and surface profiles were constructed using Nanoscope 7.0 program package. The mean surface roughness *R*_a_ at 1 × 1 μm sections and topographic histogram are also given. 1D (for one line of scan section) roughness was calculated as follows:

$$R_{a}=-A\overset{N}{\underset{j=1}{\sum }}\left|r_{j}\right|,\;\mathrm{where}\;r_{j}=z_{j}-\bar{z}.$$

Full geometry optimizations of ligands **2**, **5**, and **6** were carried out using Priroda 16 quantum chemical program at DFT (Perdew–Burke–Ernzerhof (PBE) functional and basis set of triple-ζ quality (3z)) [59,60]. DFT calculations of solvation of the ligands including their further geometry optimization with Becke 3-parameter Lee–Yang–Parr (B3LYP) functional and 6-31G basis set with the assumption of solvent (polarizable continuum model (PCM)/solvation model based on density (SMD)) were run using Gaussian 09 suit of programs [61]. Computations of compounds **5b’** and **5b’**–Ca were performed using Gaussian 16 suit of programs [62] and def-TZVP (triple-zeta valence polarization) basis set [63]. Wood–Boring Stuttgart effective core potentials [64] were implied for Ca atom. The ground-state structures were optimized at DFT using the B3LYP functional [65,66]. The D3 London dispersion correction was applied as implemented in Gaussian [67]. The electronic spectra were simulated using time-dependent DFT [68,69,70] by calculating the first 50 vertical excitations from the ground state (S_0_) equilibrium geometries with cam-B3LYP long-range-corrected functional, which yield good results for charge-transfer systems [71,72,73]. Vertical transitions were broadened with Gaussian function of full width at half maximum of 0.24 eV. The dipole length formalism was used to calculate the oscillator strengths. The excitation energies were consistently red-shifted by 0.4 eV upon cam-B3LYP calculations in order to better match experimental spectral curves.

## Figures and Tables

**Table 1:** Diffusion coefficients (*D*_s_), hydrodynamic radii (*R*_h_), and aggregation numbers (*N*_aggr_) of calixarenes **5a** and **7** in CDCl_3_ (*T* = 303 K).

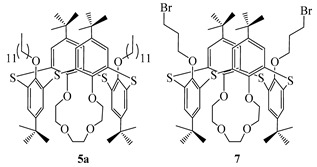	*c*, 10^−3^*M*	*D*_s_, 10^−10^ m^2^/s	*R*_h_, Å	*N*_aggr_ (from *c*_min)_
	(**5a**) 5	6.10	7.2	1.0
	(**5a**) 30	5.67	7.8	1.2
	(**7**) 5	7.61	5.8	1.0
	(**7**) 30	6.59	6.7	1.5

**Table 2:** Number-averaged PSD of thiacalix[4]crowns **2c**, **5c**, and **6c** after stirring in CH_2_Cl_2_–water.

Compound	Analyzed Phase	Metal Salt	Size, nm	Polydispersity Index
**2c**	CH_2_Cl_2_ and Water	–/CsPic	–	–
	CH_2_Cl_2_	CsNO_3_	313.1 ± 14.5	0.95 ± 0.05
	Water		298.7 ± 36.7	0.39 ± 0.08
**5c**	CH_2_Cl_2_	–	285.4 ± 32.1	1.00
		CsPic	426.4 ± 26.1	0.47 ± 0.09
		CsNO_3_	165.9 ± 34.1	0.45 ± 0.18
	Water	–	–	–
		CsPic	80.9 ± 5.6	0.46 ± 0.04
		CsNO_3_	262.7 ± 21.5	0.37 ± 0.06
**6c**	CH_2_Cl_2_	–	–	–
		CsPic	76.3 ± 15.2	0.59 ± 0.13
		CsNO_3_	259.3 ± 58.3	0.36 ± 0.23
	Water	–	331.3 ± 38.2	0.30 ± 0.05
		CsPic/CsNO_3_	–	–

**Table 3:** Monolayer characteristics of calixarenes **2**, **5**, and **6** (take-off area *A*_0_, compression modulus $C_{s}^{–1}$, calculated molecular area at vertical ($A_{⊥}$) and horizontal orientation ($A_{∥}$), surface potential (Δ*V*), surface dipole moment ($\mu_{⊥}$), and maximum absorbance (*λ*_max_)).

Ligand	*A*_0_, Å^2^	$C_{s}^{–1},\;\mathrm{mN}/m$	$A_{⊥},\;{Å}^{2}$	$A_{∥},\;{Å}^{2}$	*ΔV*, mV ^3^	$\mu_{⊥},\;D$	*λ*_max_, nm
**2a**	195 ± 6	40	117 ^1^	162	508	1.35	302
**2b**	182 ± 5	50	104 ^2^	171	517	1.37	303
**2c**	176 ± 5	37	103 ^2^	193	740	1.96	304
**5a**	201 ± 6	29	129	240	263	0.70	271
**5b**	185 ± 6	27	132	252	295	0.78	269
**5c**	177 ± 5	45	127	250	375	0.99	271
**6a**	392 ± 12	25	109	122	225	1.19	270
**6b**	467 ± 14	38	108	149	281	1.49	271
**6c**	506 ± 15	42	103	163	301	1.60	270

^1^ Calculated for *PC* conformer; ^2^ calculated for *DC* conformer; ^3^ at the molecular area of 100 Å^2^ (**2**, **5**) and 200 Å^2^ (**6**).

**Table 4:** Morphological characteristics of bare quartz and quartz coated with Langmuir–Blodgett films of ligands **2a–c**, **5a–b**, and **6a**. The concentration of the spreading solution is given in parentheses.

Compound	Number of Monolayers	*R*_a_, nm	*h*, nm	TR	*π*_transfer_, mN/m
quartz	n/a	0.20	n/a	n/a	n/a
**2a** (1 × 10^−5^ *M*)	1	0.30	n/a	0.76	10
**2b** (1 × 10^−5^ *M*)	1	0.25	n/a	0.93	12
**5a** (1 × 10^−5^ *M*)	1	2.3	n/a	0.84	6
**5b** (1 × 10^−5^ *M*)	1	2.5	n/a	1.15	12
**6a** (1 × 10^−5^ *M*)	1	1.0	n/a	0.98	10

**Table 5:** Take-off areas *A*_0_ of ligands **2a–c** and **5a–c** on the water subphase containing 1 × 10^−2^ *M* metal nitrates. Deviation of the area was less than 3%.

Ion (*r*, Å) ^1^	*A*_0_, Å^2^					
	2a	2b	2c	5a	5b	5c
–	195 ± 6	182 ± 5	176 ± 5	201 ± 6	185 ± 6	177 ± 5
Li^+^ (0.76)	213 ± 6	200 ± 6	181 ± 5	201 ± 6	188 ± 6	178 ± 5
Na^+^ (1.02)	187 ± 6	173 ± 5	172 ± 5	234 ± 7	211 ± 6	179 ± 5
K^+^ (1.38)	205 ± 6	264 ± 8	211 ± 6	215 ± 6	178 ± 6	181 ± 5
Rb^+^ (1.53)	189 ± 6	238 ± 7	207 ± 6	228 ± 7	208 ± 6	197 ± 6
Cs^+^ (1.67)	195 ± 6	208 ± 6	211 ± 6	211 ± 6	192 ± 6	212 ± 6
Mg^2+^ (0.72)	192 ± 6	186 ± 5	172 ± 5	220 ± 6	192 ± 6	183 ± 6
Ca^2+^ (1.00)	201 ± 6	189 ± 5	177 ± 5	235 ± 7	228 ± 8	199 ± 6
Sr^2+^ (1.18)	241 ± 7	200 ± 6	177 ± 5	213 ± 6	197 ± 6	181 ± 5
Ba^2+^ (1.35)	226 ± 7	180 ± 5	178 ± 5	211 ± 6	210 ± 6	184 ± 6
Pb^2+^ (1.19)	190 ± 6	214 ± 6	195 ± 6	221 ± 6	204 ± 6	179 ± 5
Ag^+^ (1.15)	221 ± 7	181 ± 5	223 ± 7	190 ± 6	191 ± 6	180 ± 5
Eu^3+^ (0.95)	203 ± 6	182 ± 5	179 ± 5	212 ± 6	182 ± 6	176 ± 5
Tb^3+^ (0.94)	203 ± 6	206 ± 6	180 ± 5	205 ± 6	193 ± 6	178 ± 5
Gd^3+^ (0.92)	204 ± 6	214 ± 6	180 ± 5	211 ± 6	179 ± 6	178 ± 5

^1^ Effective ionic radius [35].

**Table 6:** Excess Gibbs energy change Δ*G* (kJ × mol^−1^) calculated in the presence of 1 × 10^−2^ *M* metal salt solutions as subphase, with respect to pure water; *π*_0_ = 10 (**2a**; **5a,b**), 15 (**2b**, **5c**), and 20 mN/m (**2c**).

Compound	Δ*G* (metal ion)		
**2a**	1.87 (Sr^2+^)	1.36 (Ba^2+^)	1.26 (Ag^+^)
**2b**	6.52 (K^+^)	2.83 (Rb^+^)	2.46 (Gd^3+^)
**2c**	2.19 (K^+^)	3.76 (Ag^+^)	2.08 (Pb^2+^)
**5a**	1.95 (Na^+^)	1.18 (Rb^+^)	1.65 (Ca^2+^)
**5b**	1.27 (Na^+^)	5.17 (Ca^2+^)	1.13 (Ba^2+^)
**5c**	1.02 (Rb^+^)	1.94 (Cs^+^)	1.32 (Ca^2+^)

**Table 7:** Ion selectivity of thiacalixcrowns **2** and **5** in liquid and gas phases and air–water interface.

Conditions of Interaction	Ligand					
	2a	2b	2c	5a	5b	5c
Liquid–liquid interface ^1^	Ag^+^	Ag^+^	Ag^+^	Ag^+^	Ag^+^/Rb^+^	Cs^+^
Air–water interface ^2^	Sr^2+^	K^+^	Ag^+^	Na^+^	Ca^2+^	Cs^+^
Gas phase ^3^	Li^+^	Na^+^	K^+^	Li^+^/Na^+^	K^+^	Li^+^/Cs^+^

Evaluated from ^1^ extractability, ^2^ the Gibbs free energy of spreading films, and ^3^ peak abundance.

**Table 8:** CS differences (Δ*δ*, ppm) of compounds **6c**/**2c** upon the addition of AgClO_4_. Positive values indicate a downfield shift, negative values indicate an upfield shift. H_Ar_-1 stands for the proton of ArOMe/ArOH unit, H_Ar_-2 stands for the proton of Ar ring linked to crown-ether macrocycle.

Compound	H_Ar_-1	H_Ar_-2	CH_2_–1	CH_2_–2	CH_2_–3	CH_2_–4	CH_2_–5	OMe
**6c**	0.10	0.05	0.25	0.20	0.27	0.27	0.21	−0.08
**2c**	0.17	0.12	−0.41	0.08	0.16	−0.01	−0.02	n/a

## Data Availability

Not applicable.

## Supplementary Materials

Supplementary Materials can be found at https://www.mdpi.com/article/10.3390/ijms22073535/s1.

## Abbreviations

MALDI	Matrix-assisted laser desorption–ionization
CS	Chemical shift
NMR	Nuclear magnetic resonance
PC	Pinched cone
DC	Distorted cone
IR	Infrared
DLS	Dynamic light scattering
FT-PGSE	Fourier transform pulsed-gradient spin-echo
PSD	Particle size distribution
DFT	Density functional theory
TR	Transfer ratio
AFM	Atomic force microscopy
SPOT	Surface potential
UVRAS	UV/visible reflection–absorption spectroscopy
BPP-STE-LED	Bipolar pulse pair–stimulated echo-longitudinal eddy current delay
PBE	Perdew–Burke–Ernzerhof
B3LYP	Becke, 3-parameter, Lee–Yang–Parr
PCM	Polarizable continuum model
SMD	Solvation model based on density
TZVP	Triple-zeta valence polarization
