# Supplementary material for: Switching Ion Binding Selectivity of Thiacalix[4]arene Monocrowns at Liquid–Liquid and 2D-Confined Interfaces
Source: Int J Mol Sci. 2021 Mar 29;22(7):3535. doi: 10.3390/ijms22073535 (PMC8038083; doi:10.3390/ijms22073535)
Supplement: Supplementary file 1 [file ijms-22-03535-s001.pdf]

Switching Ion Binding Selectivity of Thiacalix[4]arene Monocrowns at Liquid–Liquid and 2D-Confined Interfaces

Anton Muravev[1,]*, Ayrat Yakupov[2], Tatiana Gerasimova[1], Ramil Nugmanov[2], Ekaterina Trushina[3], Olga Babaeva[1], Guliya Nizameeva[4], Viktor Syakaev[1], Sergey Katsyuba[1], Sofiya Selektor[5], Svetlana Solovieva[2], and Igor Antipin[2]

[1]Arbuzov Institute of Organic and Physical Chemistry, FRC Kazan Scientific Center, Russian Academy of Sciences, Kazan, 420088 Russia

[2]Kazan Federal University, Kazan, 420008 Russia

[3]School of Chemistry, University of Glasgow, University Avenue, G12 8QQ Glasgow, United Kingdom

[4]Kazan National Research Technological University, Kazan, 420015 Russia

[5]Frumkin Institute of Physical Chemistry and Electrochemistry, Russian Academy of Sciences, Moscow, 119071 Russia

Corresponding author e-mail: antonm@iopc.ru

Table of contents

Structures of the compounds 2

Physical characteristics of compounds **2**, **5**, and **6** 3

Gas-phase complexation data of thiacalixcrown receptors 13

Dynamic light scattering data of thiacalixcrown receptors 15

Computational data of thiacalix[4]monocrown-ethers 16

Langmuir monolayer measurements of thiacalix[4]crown-ethers 17

* Corresponding author. Tel.: +7-843-273-9365; fax: +7-843-273-1872; e-mail: antonm@iopc.ru

Structures of the compounds

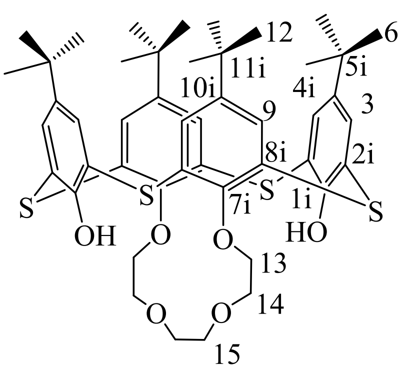

2a

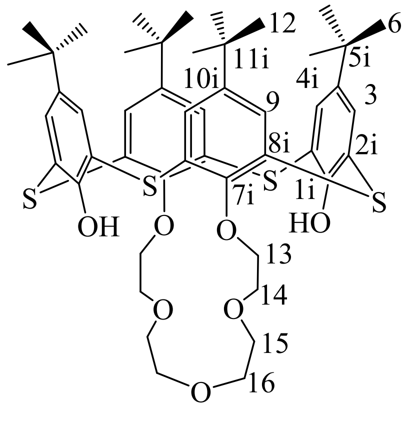

2b

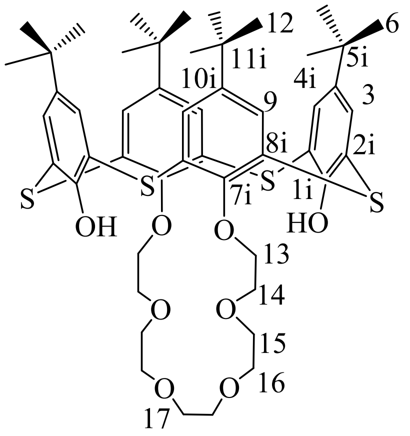

2c

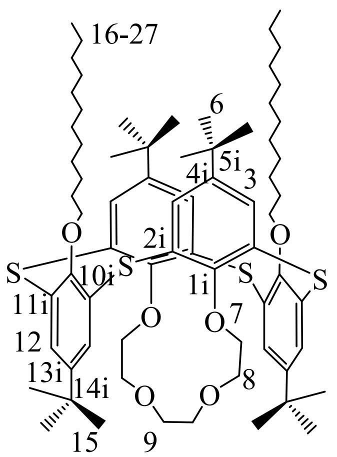

5a

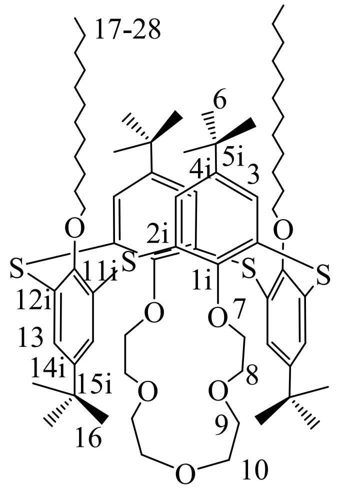

5b

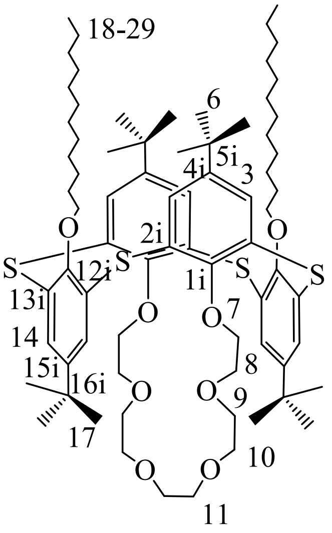

5c

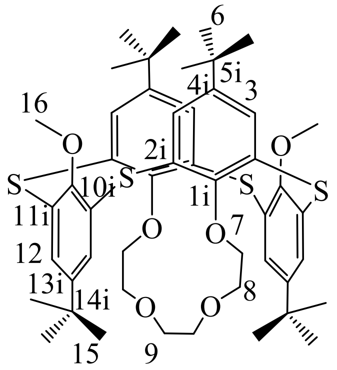

6a

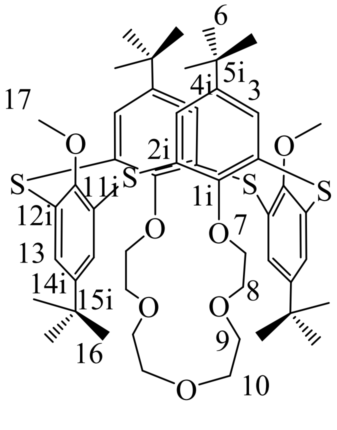

6b

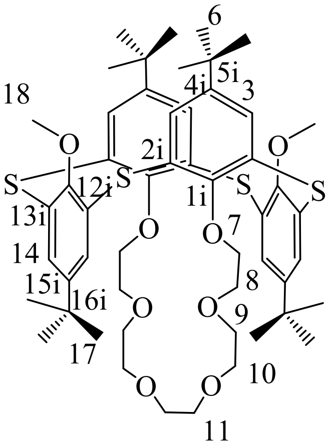

6c

Scheme S1. Structures of (thia)calix[4]arene-monocrown ethers and numbering of carbon atoms.

Physical characteristics of compounds 2, 5, and 6

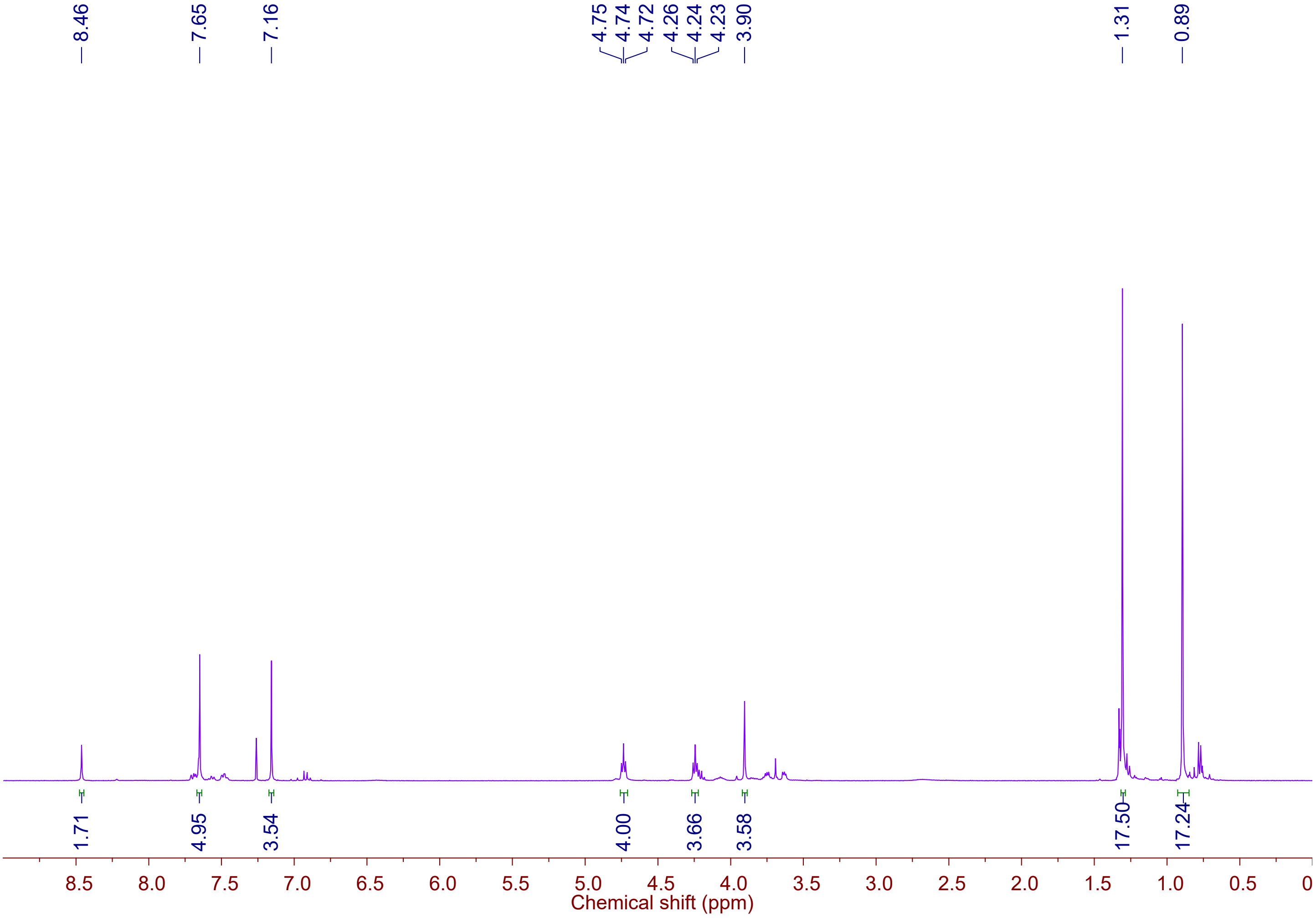

Fig. S1. ^{1}H NMR spectrum of compound **2a** ($CDCl_3$, 400 MHz, 303 K).

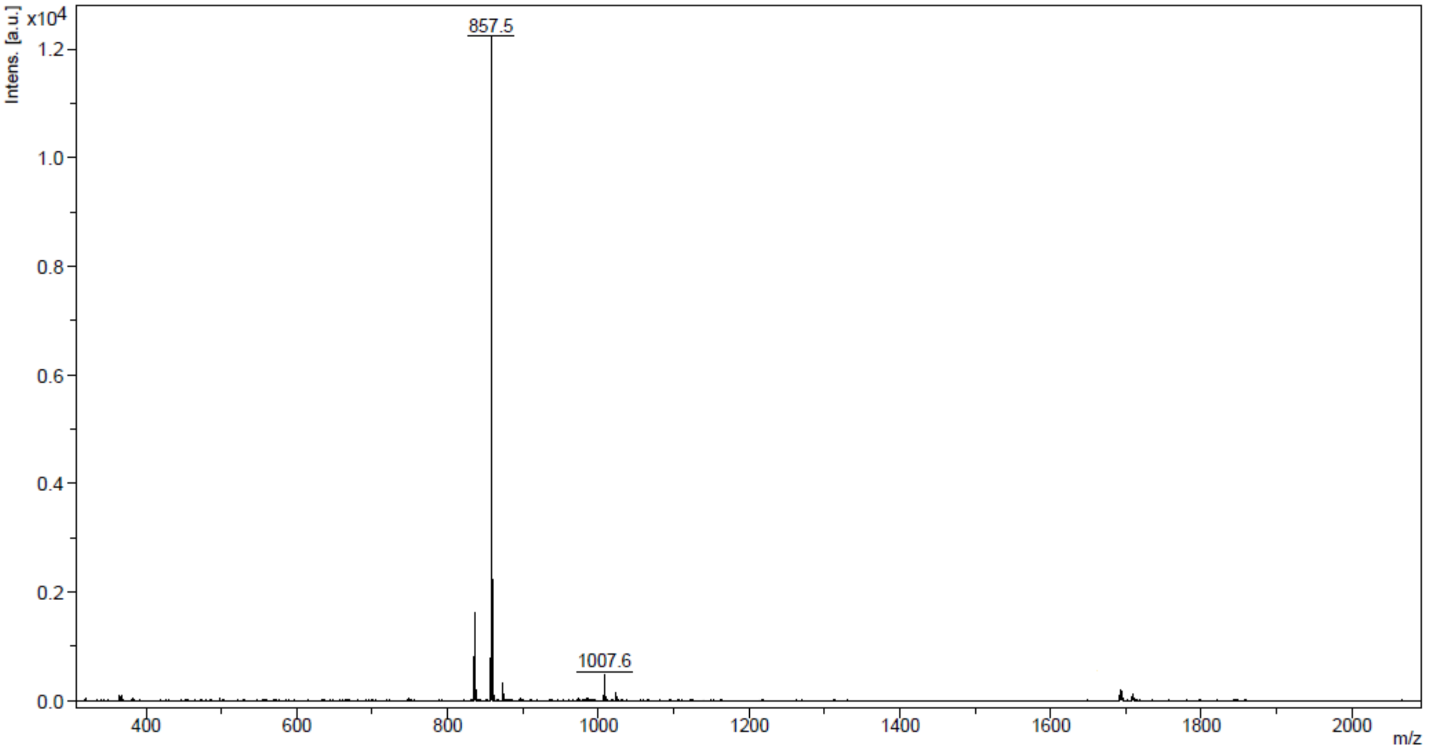

Fig. S2. MALDI mass spectrum of compound **2a** (*p*-nitroaniline matrix).

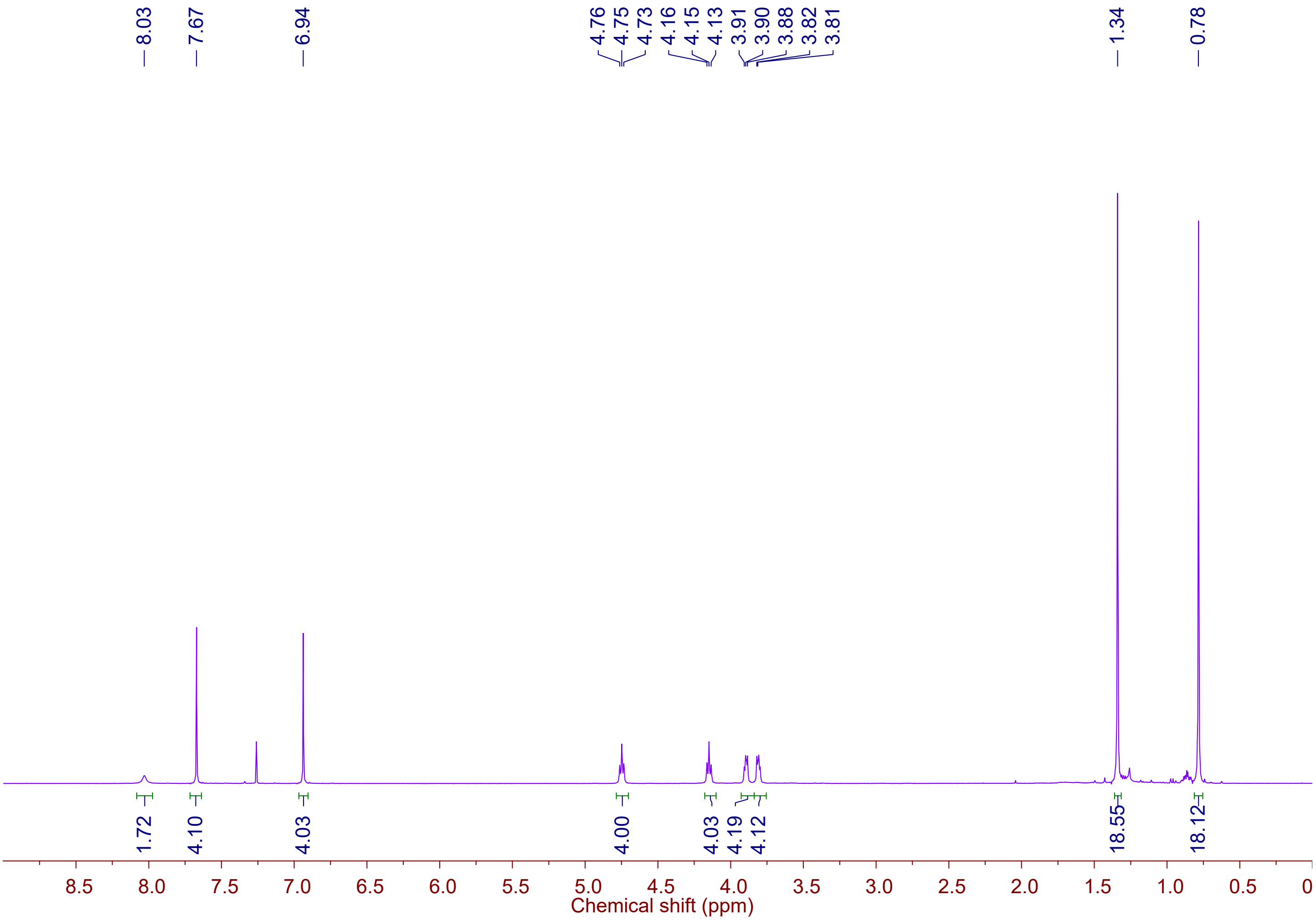

Fig. S3. ^{1}H NMR spectrum of compound **2b** ($CDCl_3$, 400 MHz, 303 K).

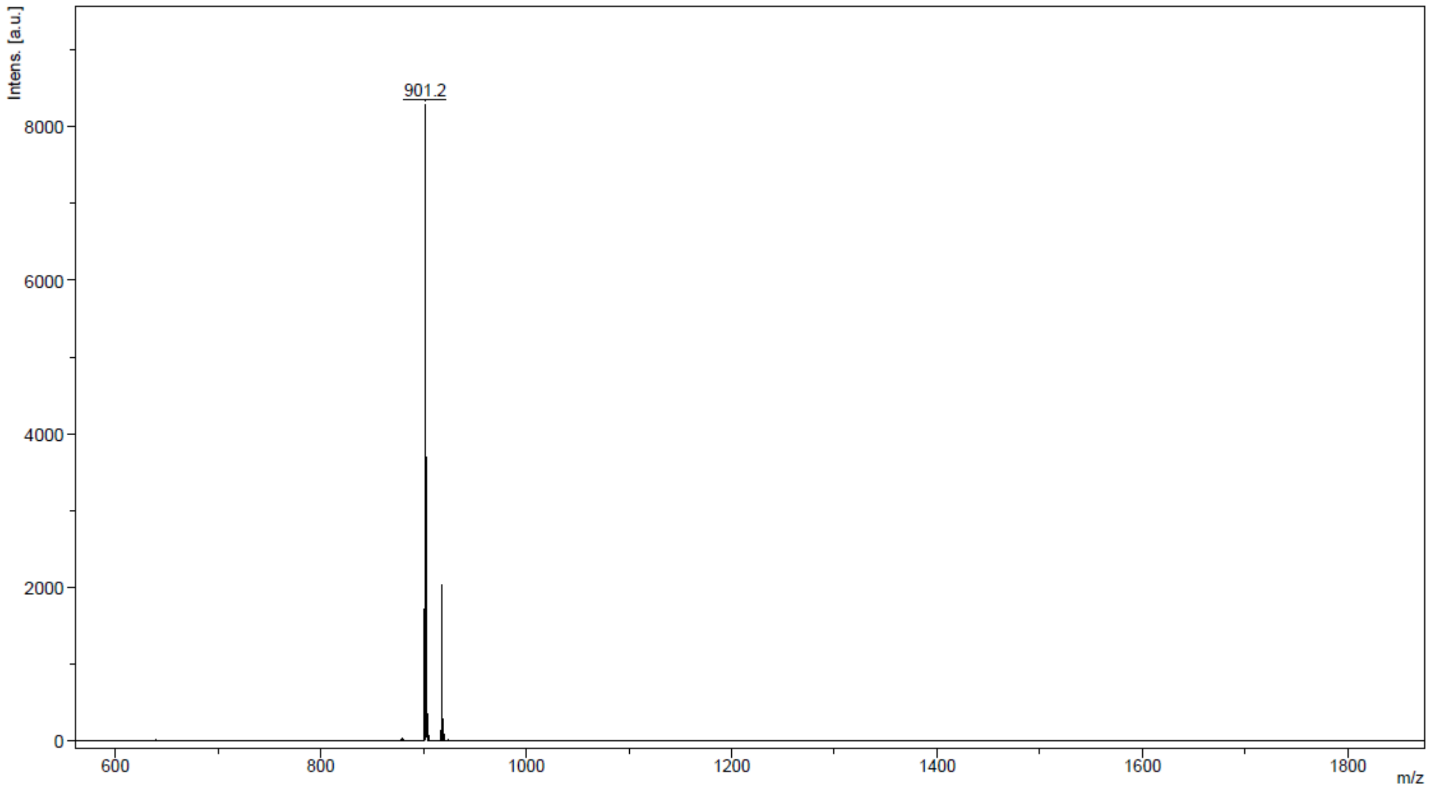

Fig. S4. MALDI mass spectrum of compound **2b** (*p*-nitroaniline matrix).

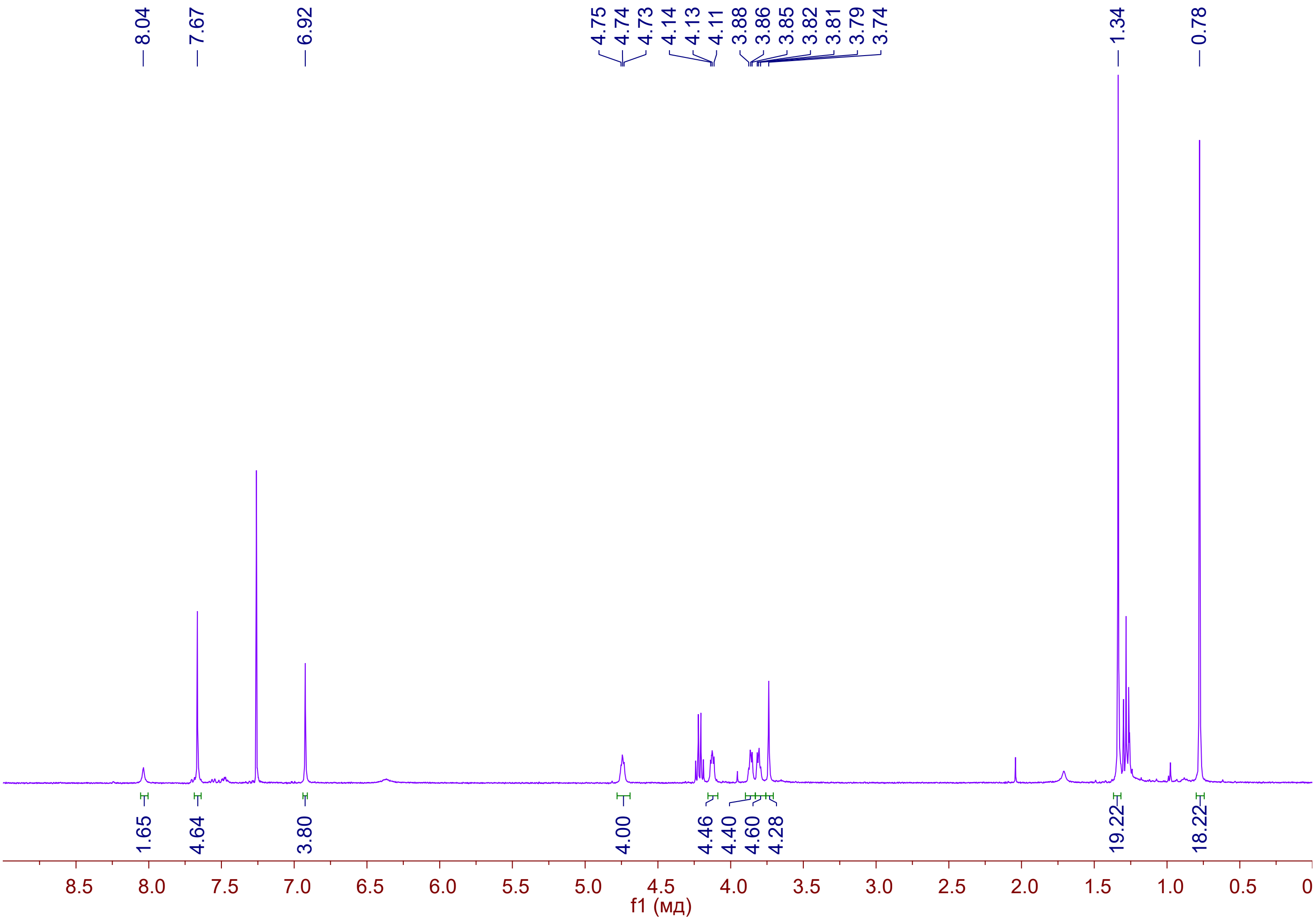

Fig. S5. ^{1}H NMR spectrum of compound **2c** ($CDCl_3$, 400 MHz, 303 K).

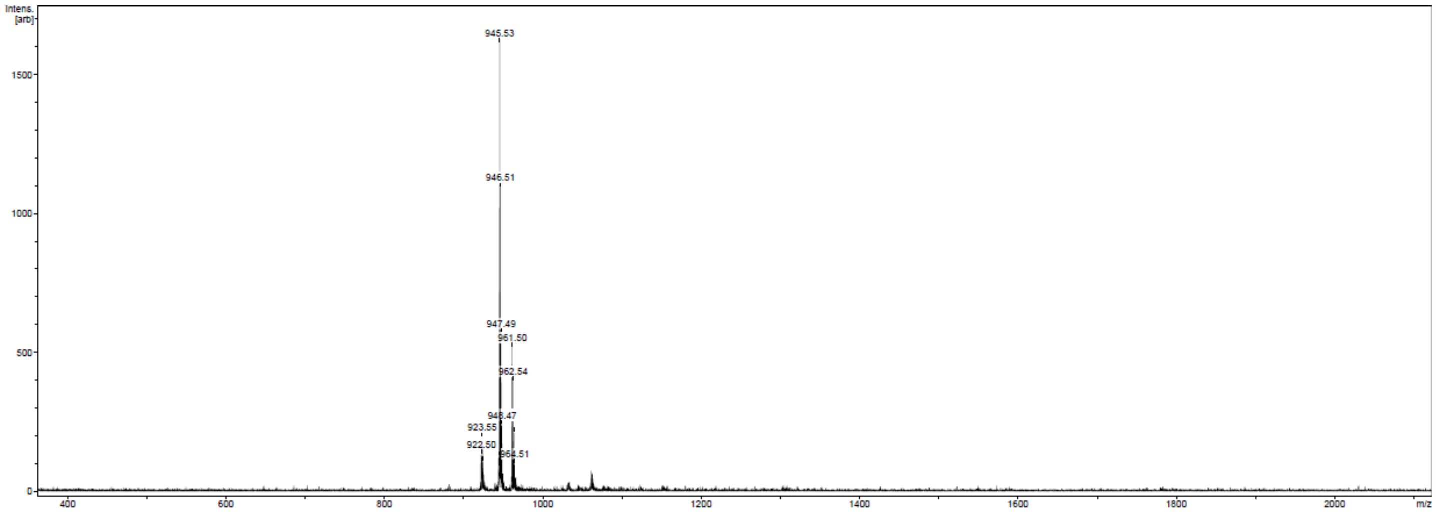

Fig. S6. MALDI mass spectrum of compound **2c** (*p*-nitroaniline matrix).

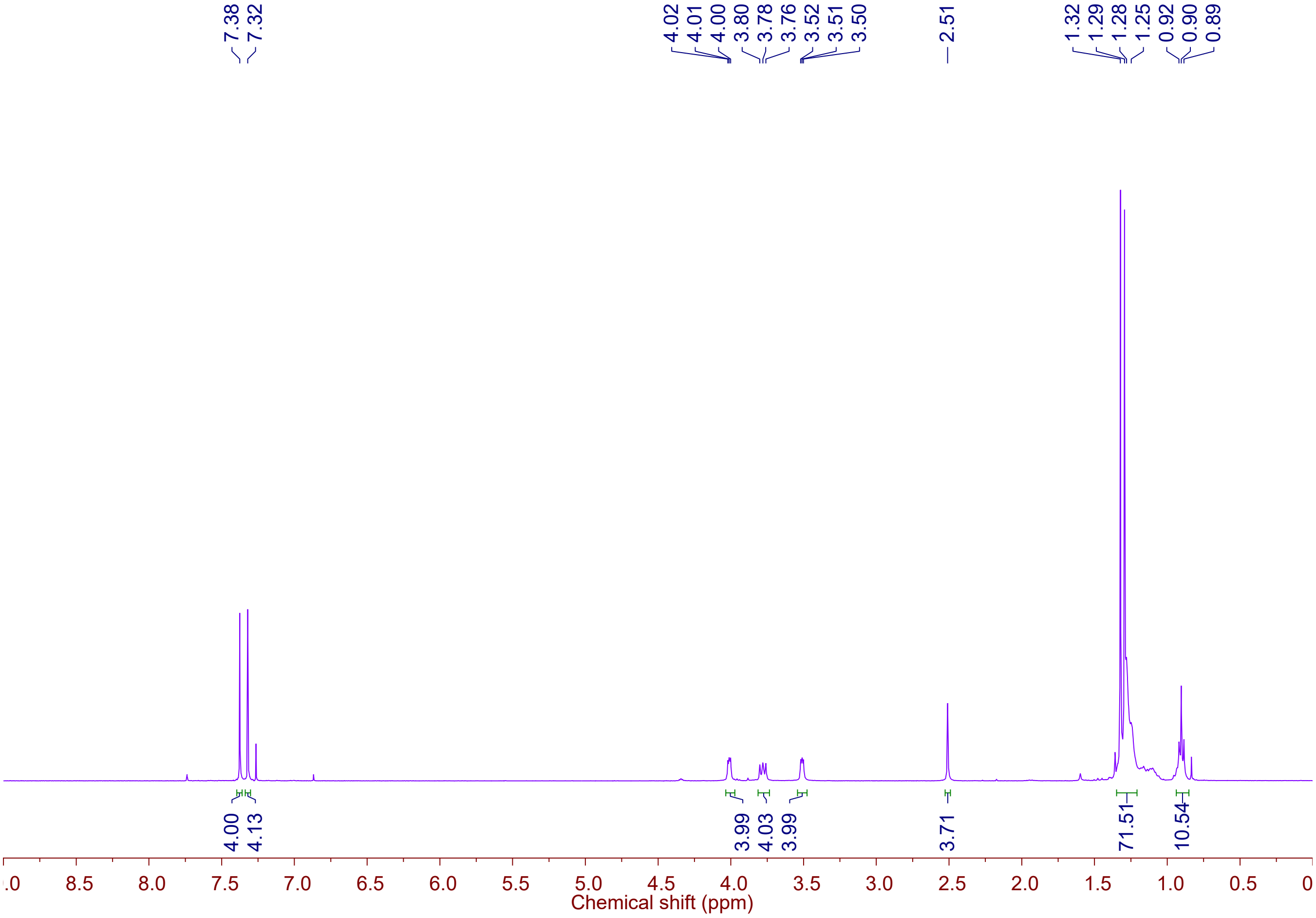

Fig. S7. ^{1}H NMR spectrum of compound **5a** ($CDCl_3$, 400 MHz, 303 K).

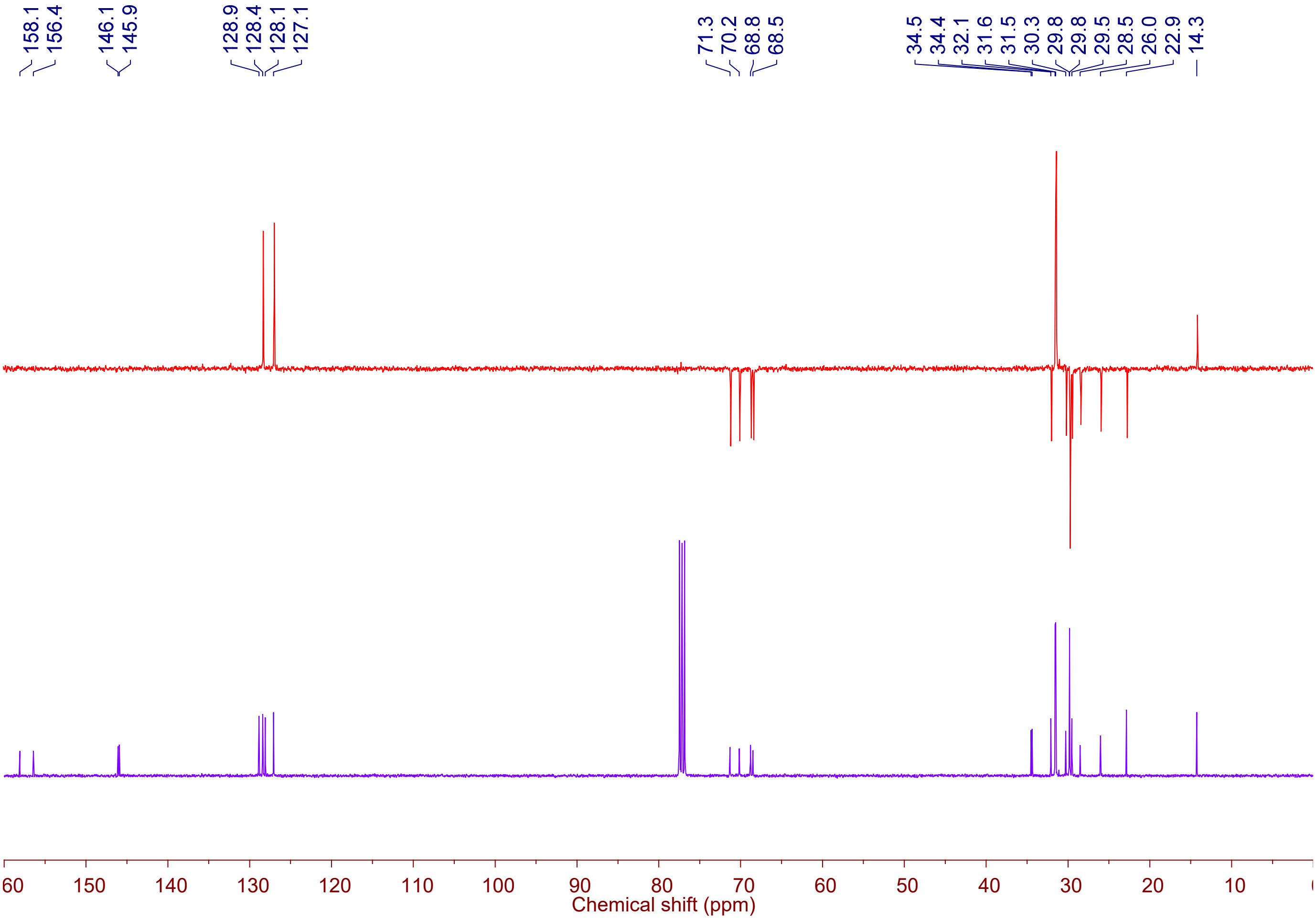

Fig. S8. ^{13}C NMR spectrum and DEPT-135 experiment of compound **5a** ($CDCl_3$, 100 MHz, 303 K).

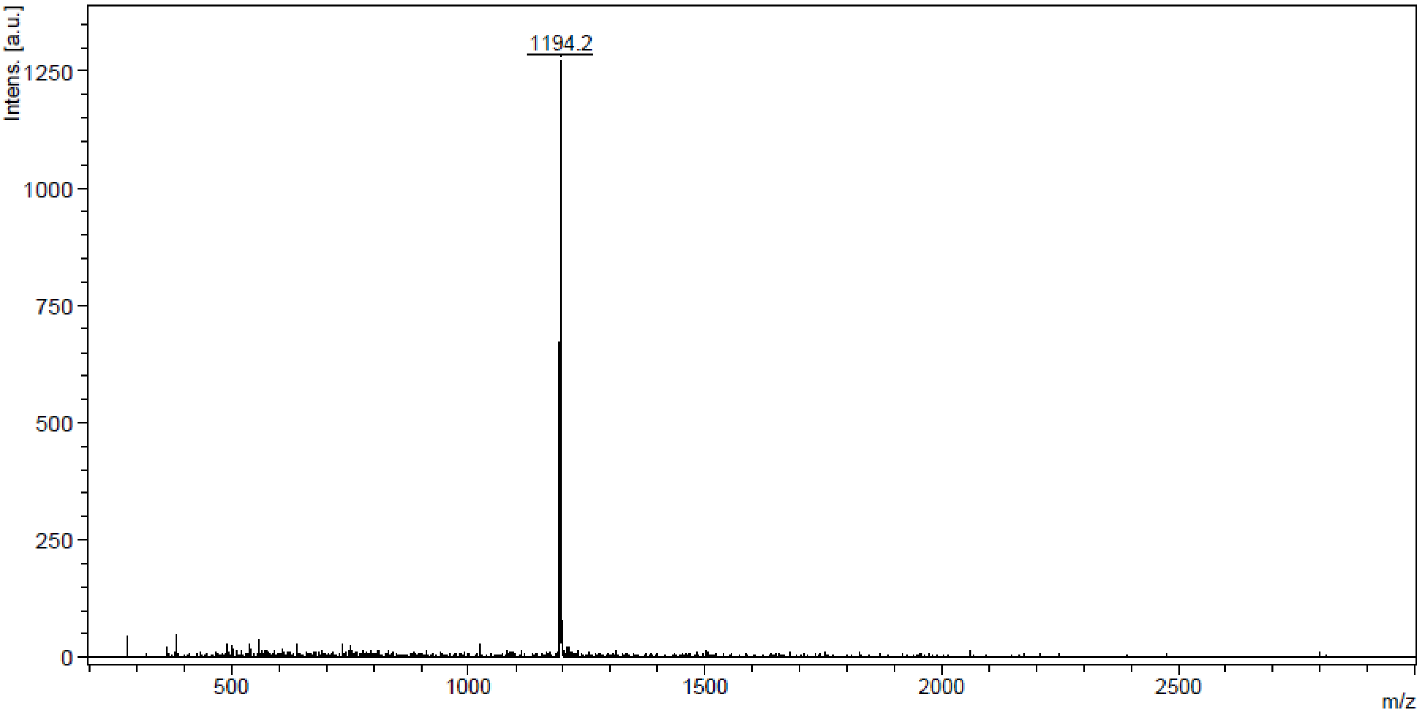

Fig. S9. MALDI mass spectrum of compound **5a** (*p*-nitroaniline matrix).

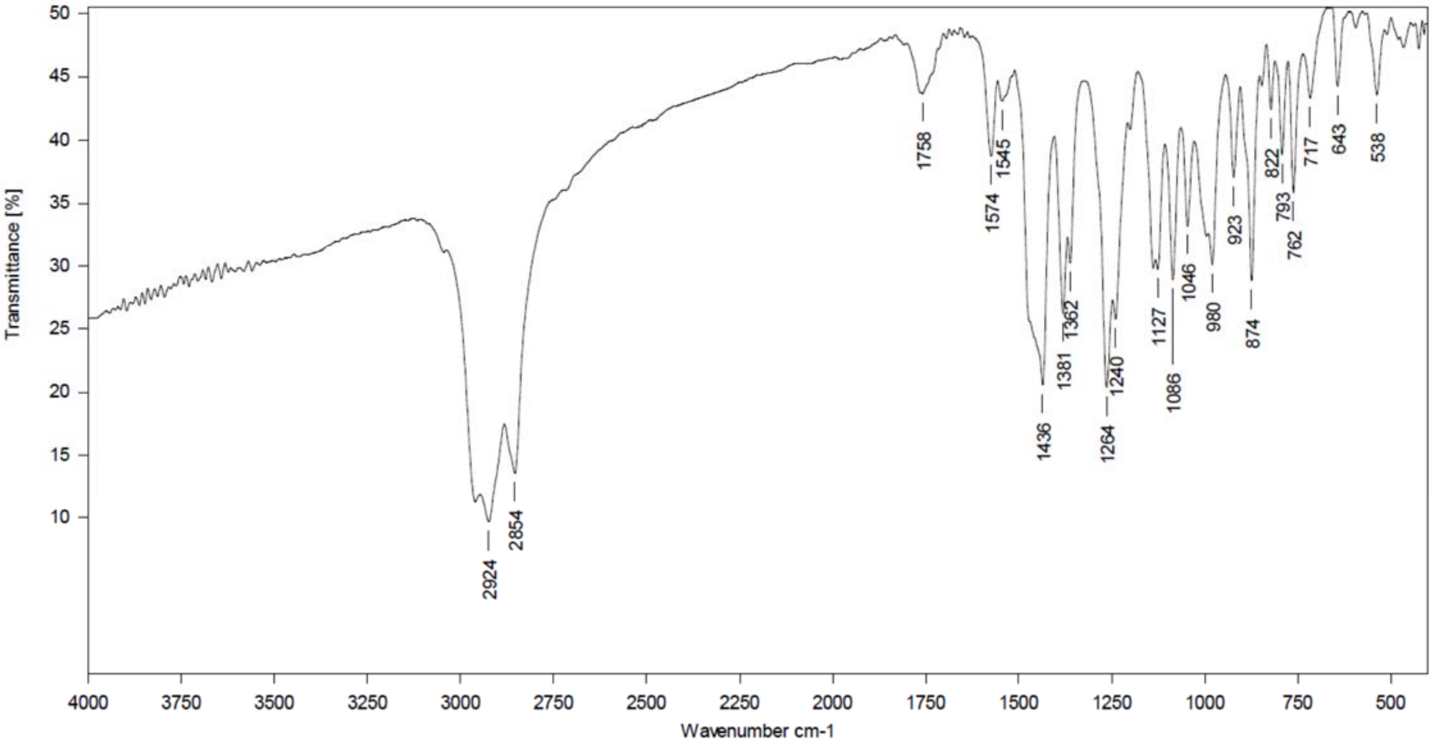

Fig. S10. IR spectrum of compound **5a** (KBr).

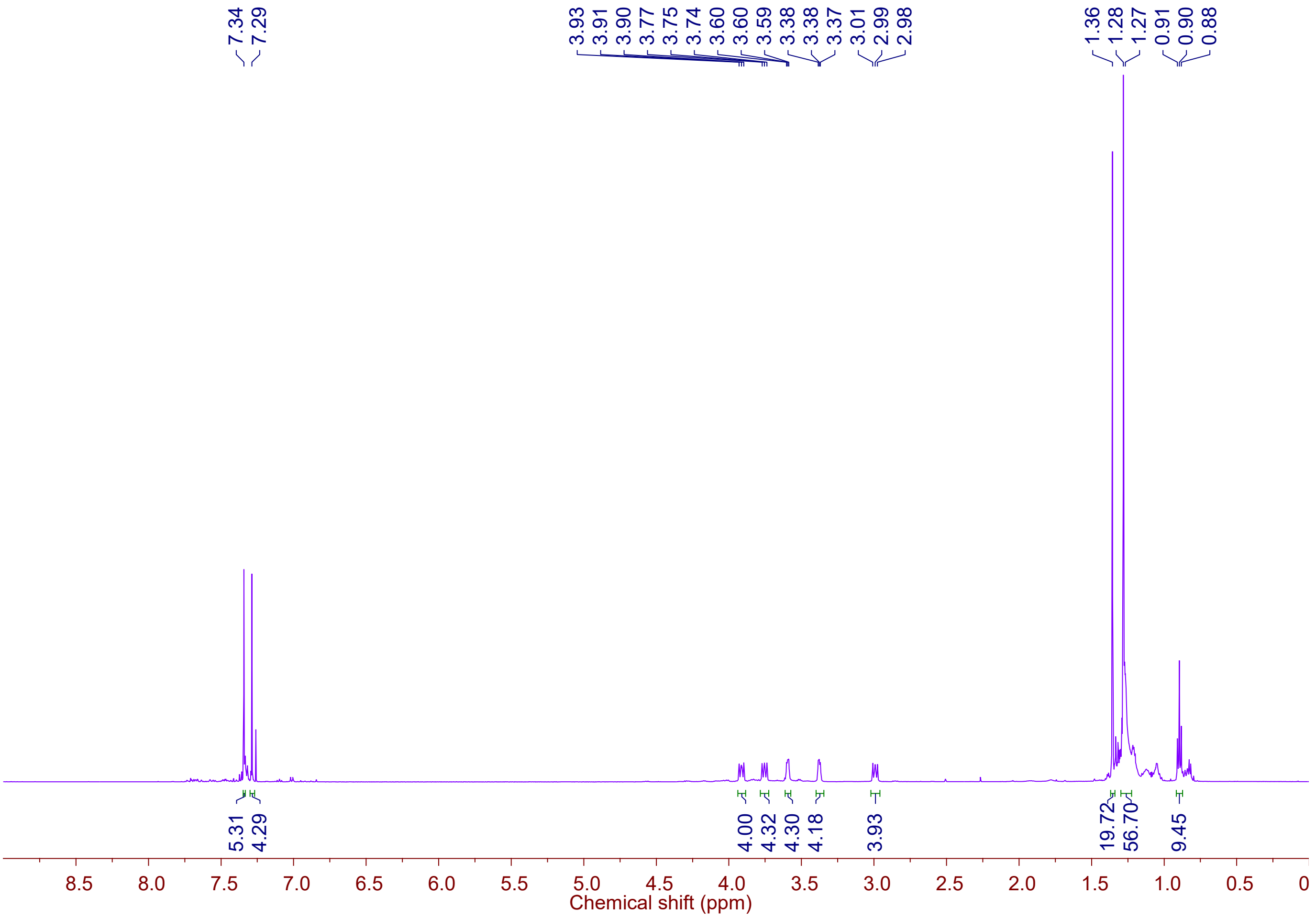

Fig. S11. ^{1}H NMR spectrum of compound **5b** ($CDCl_3$, 500 MHz, 303 K).

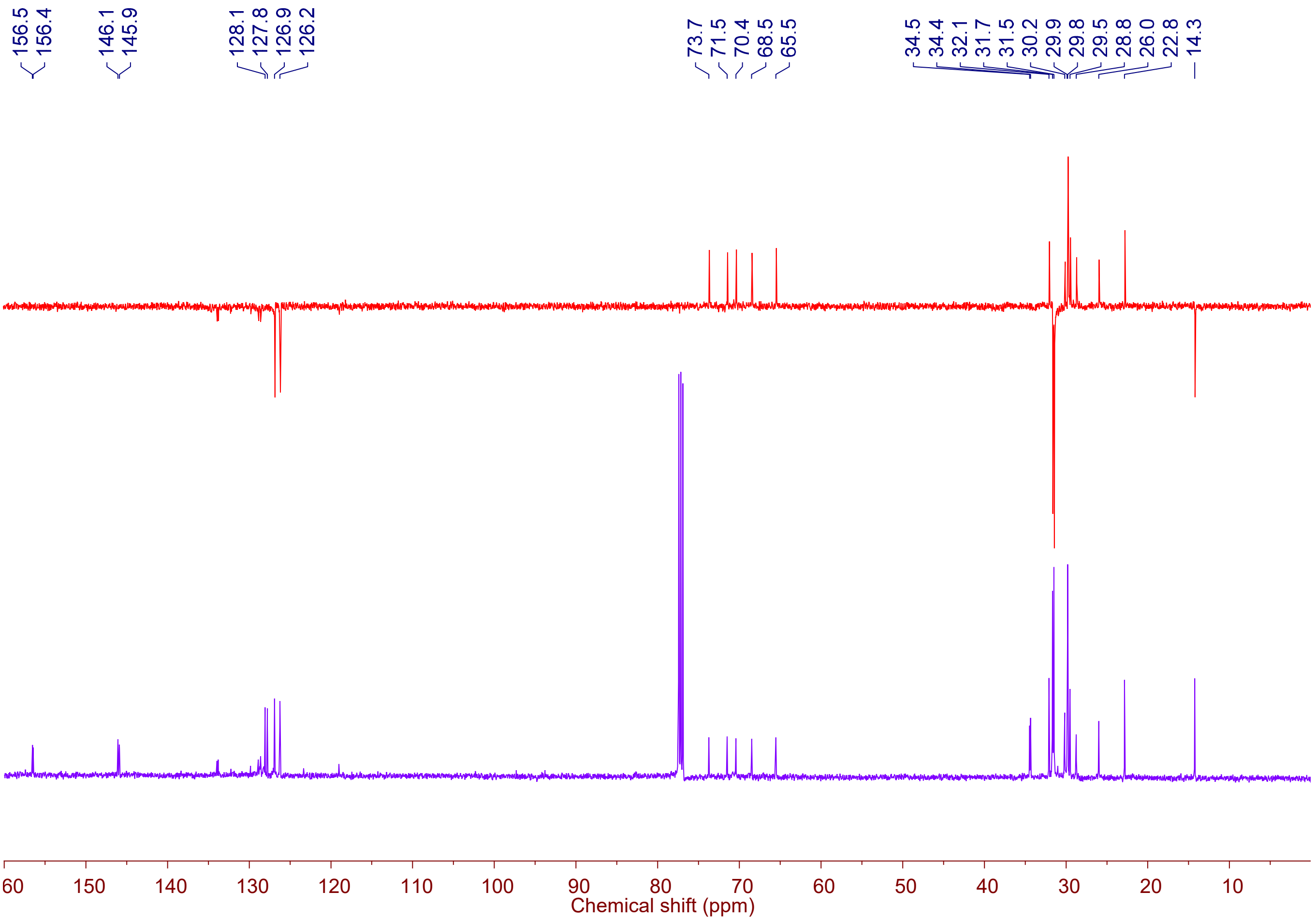

Fig. S12. ^{13}C NMR spectrum and DEPT-135 experiment of compound **5b** ($CDCl_3$, 126 MHz, 303 K).

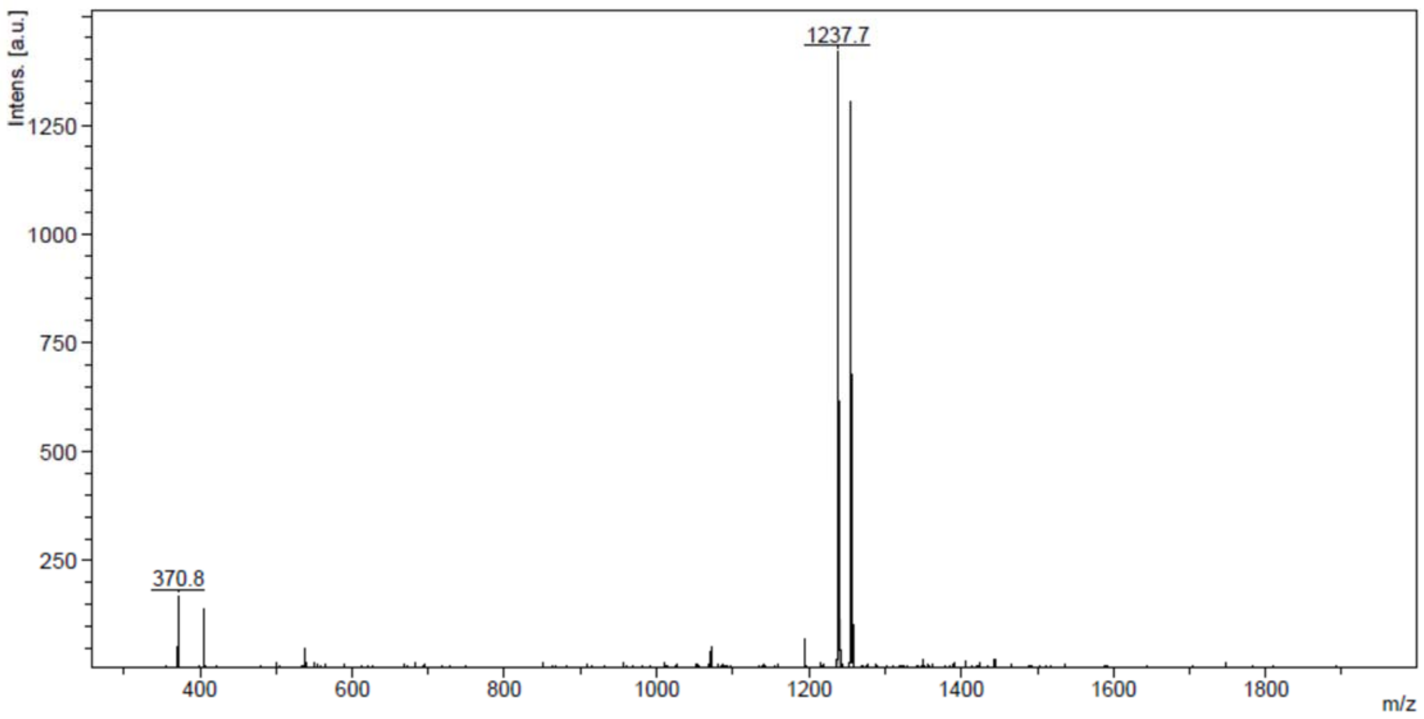

Fig. S13. MALDI mass spectrum of compound **5b** (*p*-nitroaniline matrix).

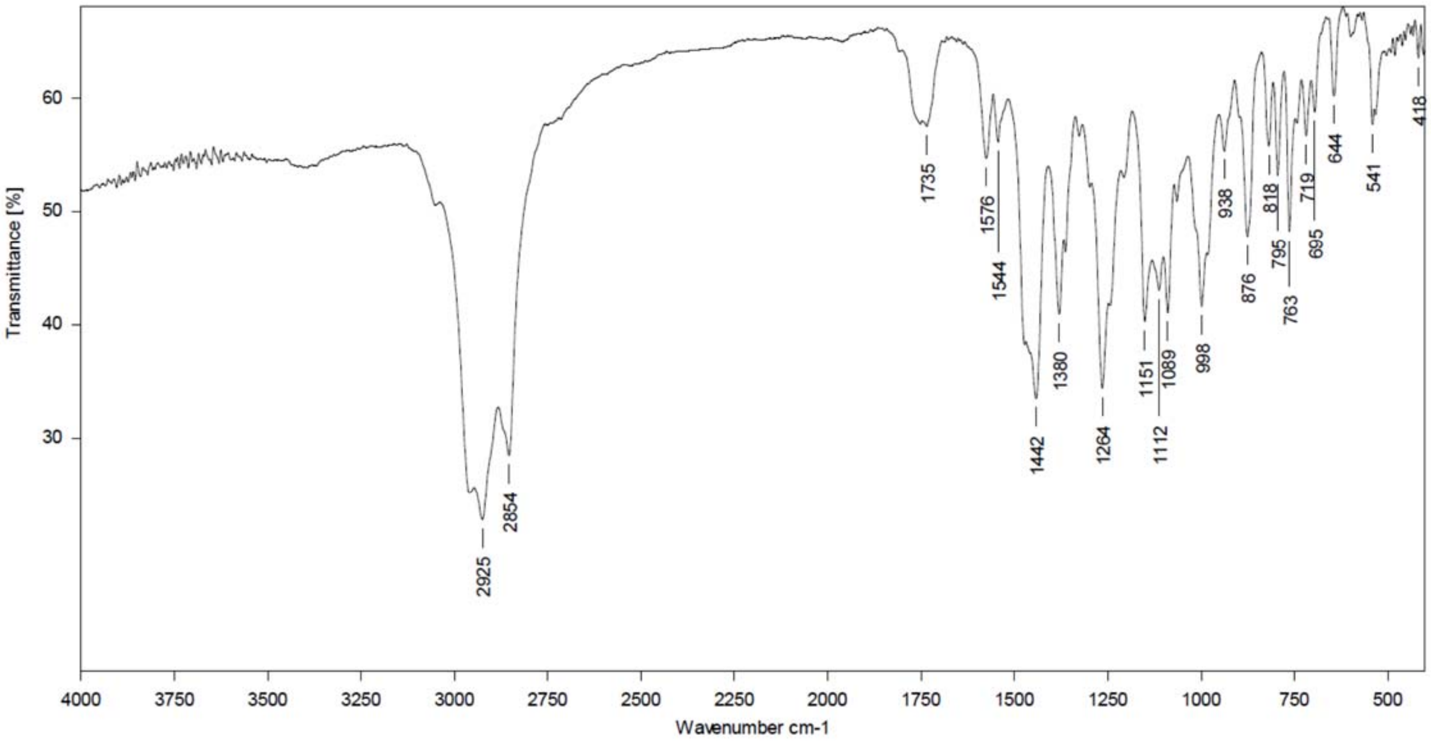

Fig. S14. IR spectrum of compound **5b** (KBr).

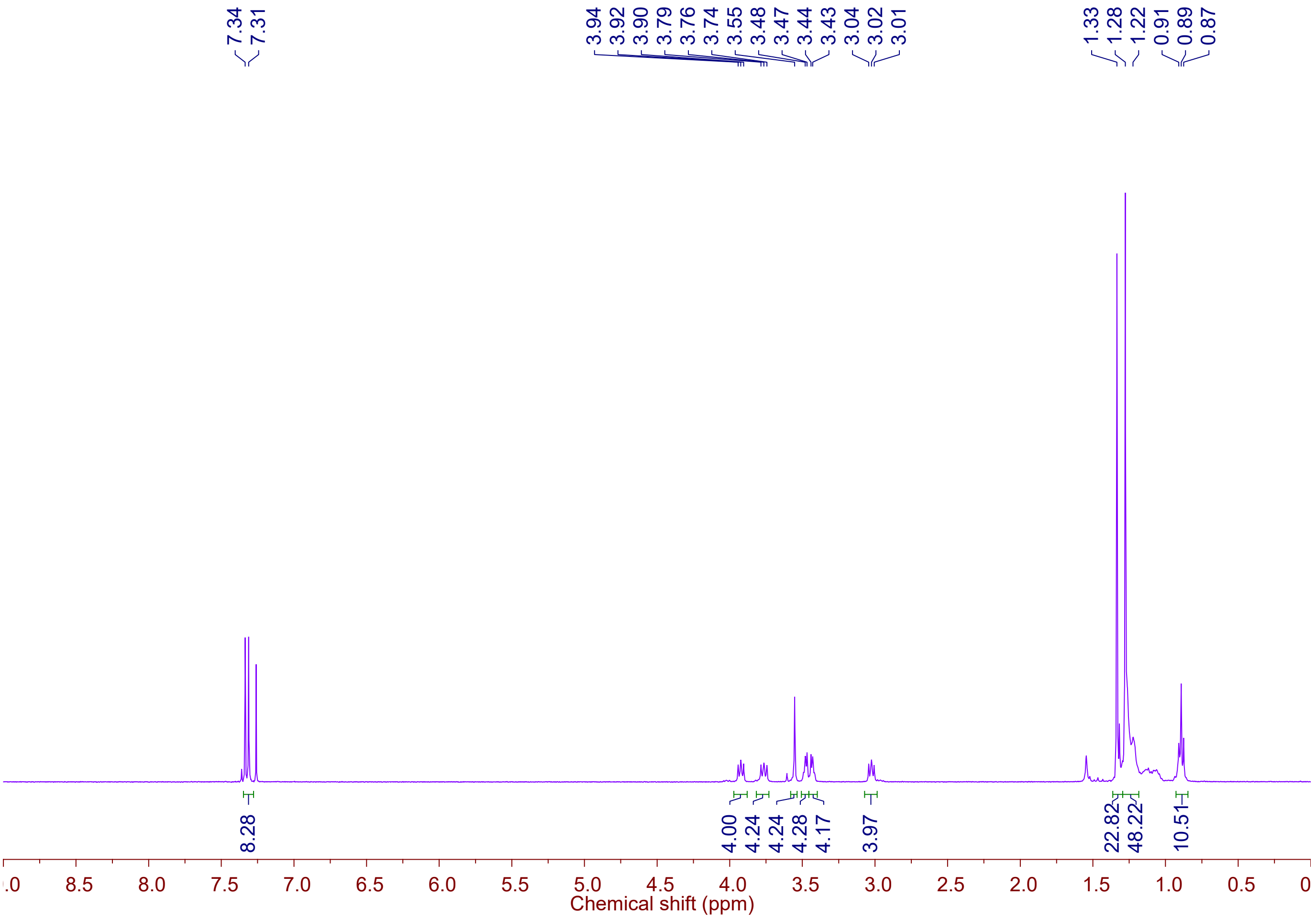

Fig. S15. ^{1}H NMR spectrum of compound **5c** ($CDCl_3$, 400 MHz, 303 K).

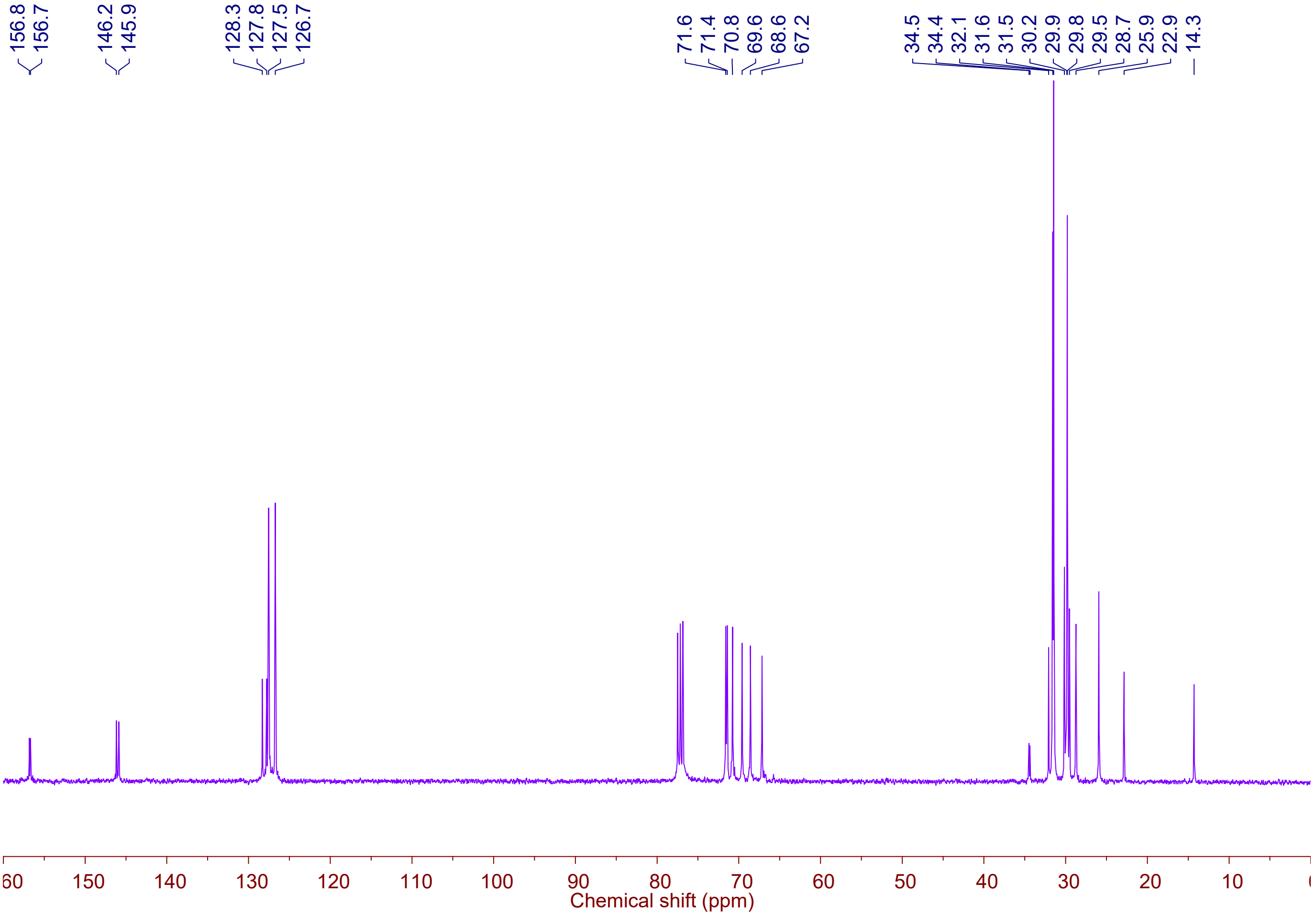

Fig. S16. ^{13}C NMR spectrum of compound **5c** ($CDCl_3$, 100 MHz, 295 K).

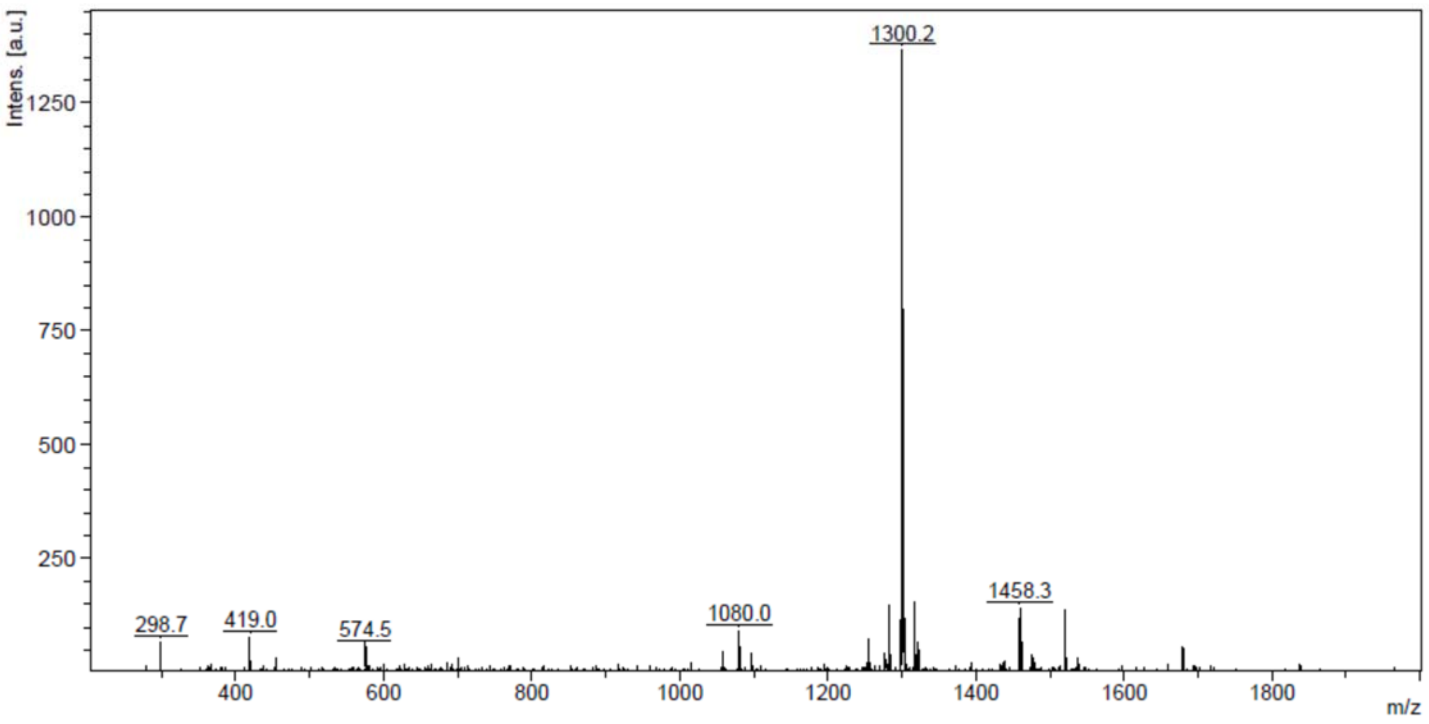

Fig. S17. MALDI mass spectrum of compound **5c** (*p*-nitroaniline matrix).

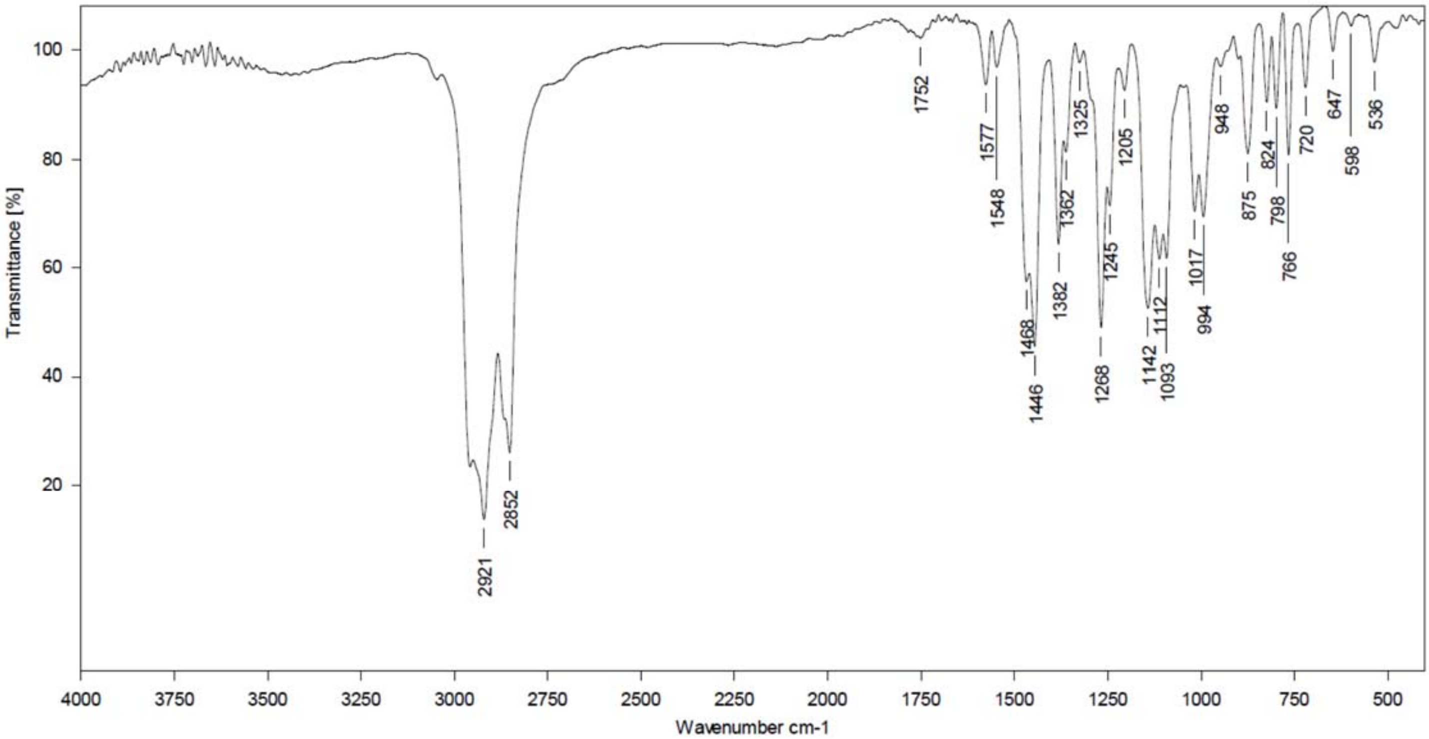

Fig. S18. IR spectrum of compound **5c** (KBr).

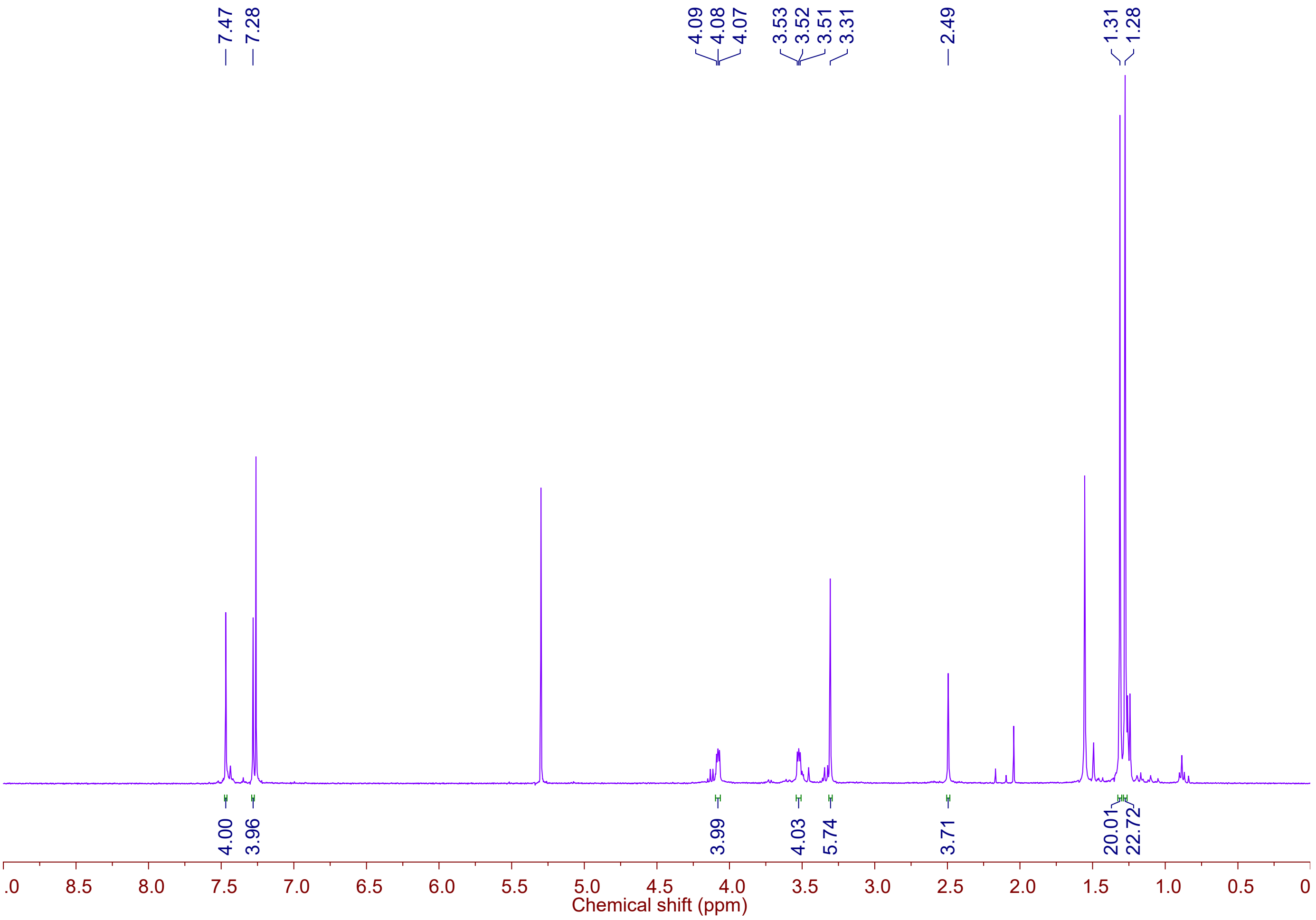

Fig. S19. ^{1}H NMR spectrum of compound **6a** ($CDCl_3$, 400 MHz, 303 K).

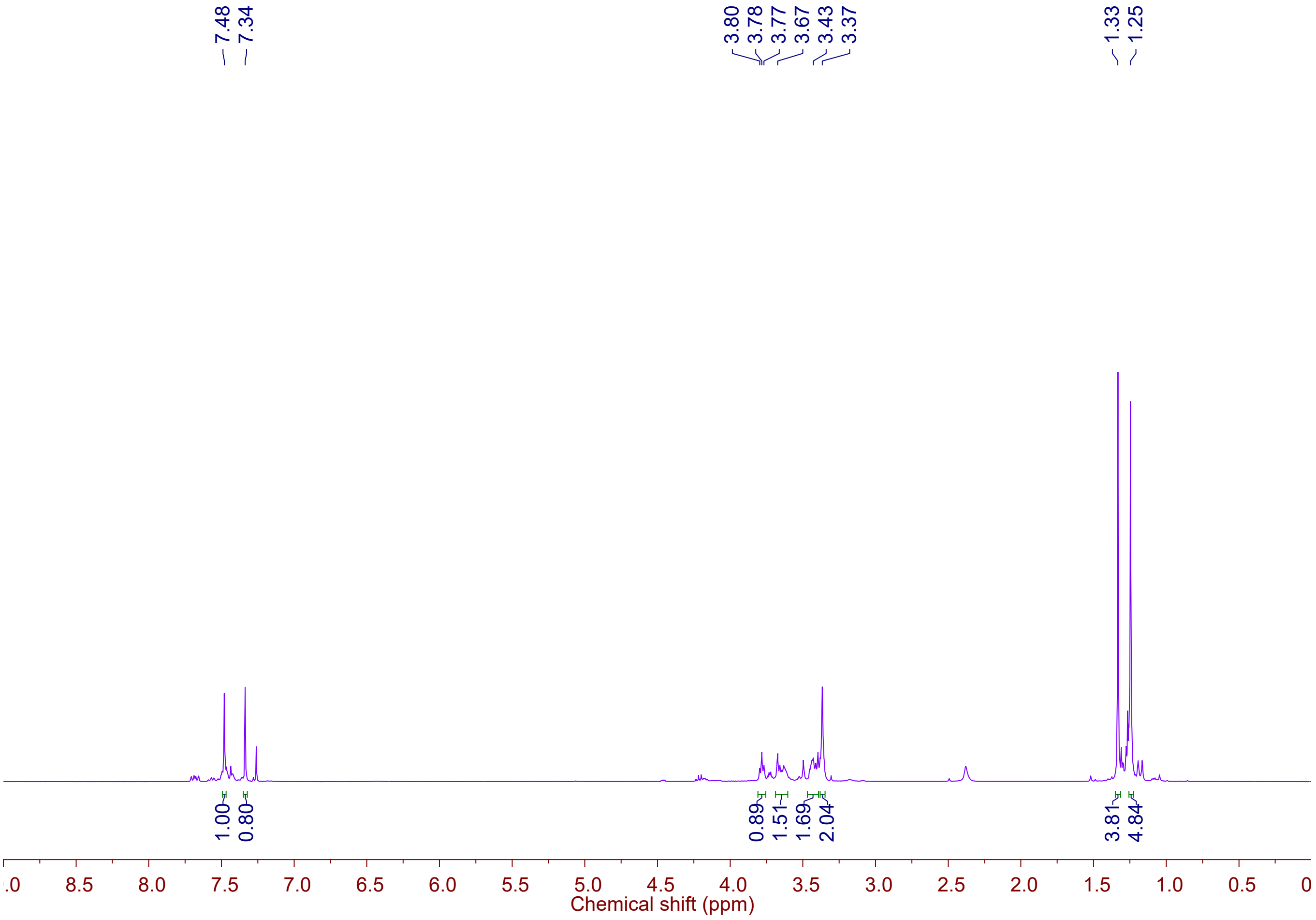

Fig. S20. ^{1}H NMR spectrum of compound **6b** ($CDCl_3$, 400 MHz, 303 K).

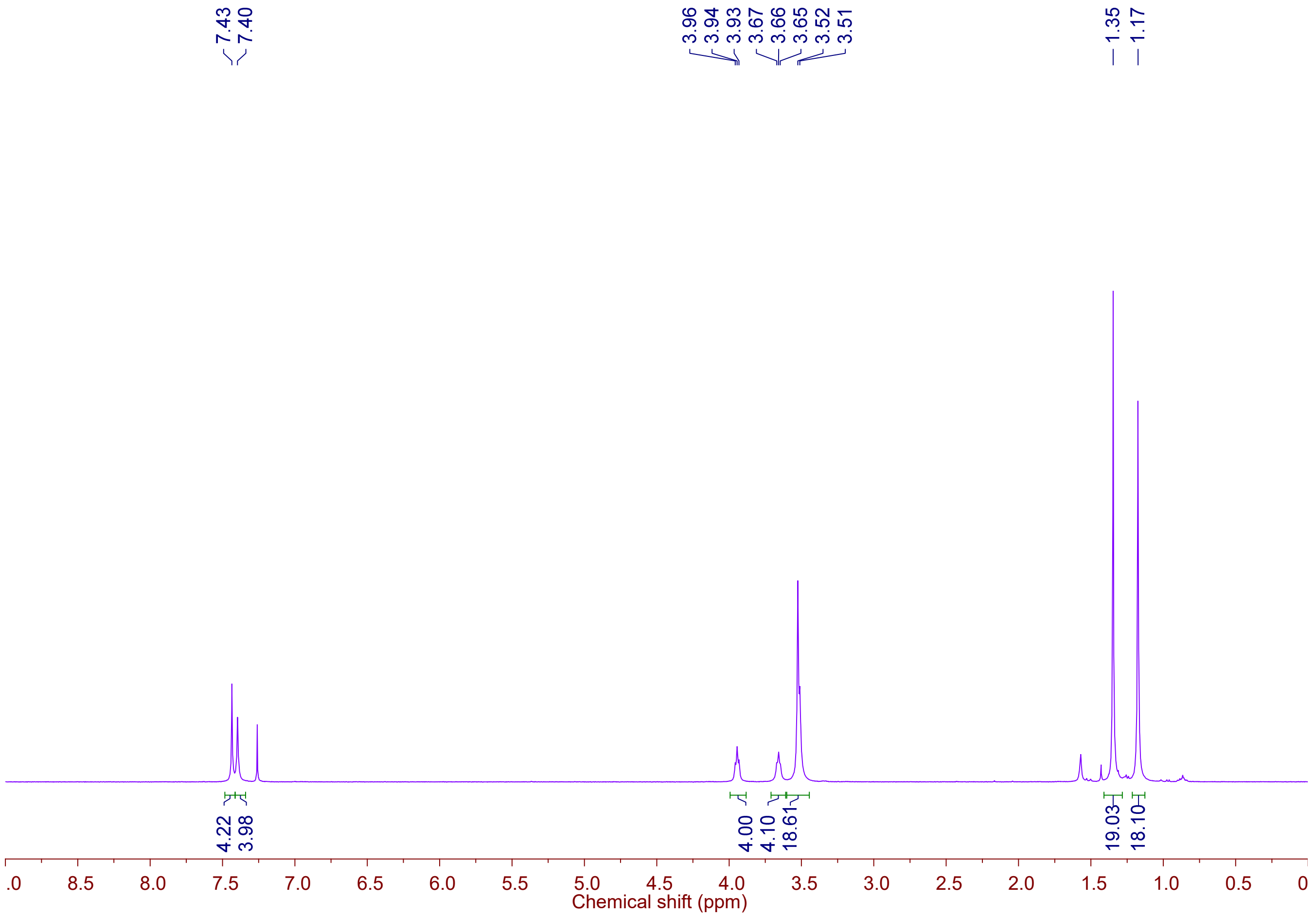

Fig. S21. ^{1}H NMR spectrum of compound **6c** ($CDCl_3$, 400 MHz, 303 K).

Gas-phase complexation data of thiacalixcrown receptors

Table S1. MALDI mass peaks and relative abundancies of thiacalixcrowns **2** and **5** mixed with nitrate salts

Ion	2a	2b	2c	5a	5b	5c
Li^+	$[M+Li]^+$ 100%<br>$[M+2Li-H]^+$ 6.4%	$[M+Li]^+$ 100%<br>$[M+2Li-H]^+$ 28%	$[M+Li]^+$ 23%<br>$[M+2Li-H]^+$ 100%	$[M+Li]^+$ 100%<br>$[M+Na]^+$ 3.1%	$[M+Li]^+$ 100%<br>$[M+Na]^+$ 3%<br>$[M+K]^+$ 8%	$[M+Li]^+$ 100%
Na^+	$[M+H]^+$ 6%<br>$[M+Na]^+$ 100%	$[M+H]^+$ <1%<br>$[M+Na]^+$ 100%<br>$[M+K]^+$ 30%	$[M+Na]^+$ 100%<br>$[M+2Na-H]^+$ 1.6%<br>$[M+K]^+$ 29%	$[M+Na]^+$ 100%<br>$[M+K]^+$ 1.2%	$[M+H]^+$ 1.5%<br>$[M+Na]^+$ 100%<br>$[M+K]^+$ 37%	$[M+Na]^+$ 100%<br>$[M+K]^+$ 4.6%
K^+	$[M+H]^+$ 66.5%<br>$[M+Na]^+$ 63%<br>$[M+K]^+$ 100%	$[M+Na]^+$ 3.8%<br>$[M+K]^+$ 100%<br>$[M+2K-H]^+$ 1.4%	$[M+Na]^+$ 1.2%<br>$[M+K]^+$ 100%<br>$[M+2K-H]^+$ 3%	$[M+Na]^+$ 100%<br>$[M+K]^+$ 3.3%	$[M+K]^+$ 100%	$[M+H]^+$ 3.7%<br>$[M+Na]^+$ 7%<br>$[M+K]^+$ 100%
Rb^+	$[M+H]^+$ 78%<br>$[M+Na]^+$ 78%<br>$[M+K]^+$ 22%<br>$[M+Rb]^+$ 100%	$[M+Na]^+$ 34%<br>$[M+K]^+$ 31%<br>$[M+Rb]^+$ 100%<br>$[M+2Rb-H]^+$ <1%	$[M+K]^+$ 2.7%<br>$[M+Rb]^+$ 100%<br>$[M+2Rb-H]^+$ <1%	$[M+Na]^+$ 100%<br>$[M+K]^+$ 2%<br>$[M+Rb]^+$ 4.5%	$[M+H]^+$ <1%<br>$[M+Na]^+$ <1%<br>$[M+K]^+$ 5.3%<br>$[M+Rb]^+$ 100%	$[M+H]^+$ <1%<br>$[M+Na]^+$ <1%<br>$[M+K]^+$ <1%<br>$[M+Rb]^+$ 100%
Cs^+	$[M+H]^+$ (7.2%)<br>$[M+Na]^+$ (99%)<br>$[M+K]^+$ (6.6%)<br>$[M+Cs]^+$ (100%)	$[M+H]^+$ 5%<br>$[M+Na]^+$ 100%<br>$[M+K]^+$ 18%<br>$[M+Cs]^+$ 78%<br>$[M+2Cs-H]^+$ <1%	$[M+Na]^+$ 7.3%<br>$[M+K]^+$ 2%<br>$[M+Cs]^+$ 100%<br>$[M+2Cs-H]^+$ <1%	$[M+Na]^+$ 100%<br>$[M+K]^+$ 3.5%<br>$[M+Cs]^+$ 4.5%	$[M+H]^+$ 5.5%<br>$[M+Na]^+$ 19%<br>$[M+K]^+$ 100%<br>$[M+Cs]^+$ 32%	$[M+Cs]^+$ 100%

Mg^{2+}	$[M+H]^+$ 10%<br>$[M+K]^+$ 1.3%<br>$[M+Mg-H]^+$/$[M+Na]^+$ 100%<br>$[M+MgNO_3+Mg-2H]^+$ 4%	$[M+K]^+$ 4.7%<br>$[M+Mg-H]^+$/$[M+Na]^+$ 100%<br>$[M+MgNO_3+Mg-2H]^+$ 26%	$[M+H]^+$ 80%<br>$[M+Na]^+$ 100%<br>$[M+K]$ 66%	$[M+Na]^+$ 100%	$[M+H]^+$ 1.4%<br>$[M+Na]^+$ 16%<br>$[M+K]^+$ 100%	$[M+H]^+$ 82%<br>$[M+Na]^+$ 100%<br>$[M+K]^+$ 88%<br>$[M+MgNO_3]^+$ 55%
Ca^{2+}	$[M+H]^+$ 22.5%<br>$[M+Na]^+$ 1.2%<br>$[M+Ca-H]^+$/$[M+K]^+$ 100%	$[M+Na]^+$ (4%)<br>$[M+Ca-H]^+$/$[M+K]^+$ 100%	$[M+H]^+$ 27%<br>$[M+Na]^+$ 54%<br>$[M+Ca-H]^+$/$[M+K]^+$ 100%<br>$[M+CaNO_3]^+$ 12%	$[M+Na]^+$ 100%<br>$[M+K]^+$ 3.4%<br>$[M+CaNO_3]^+$ 7.5%	$[M+H]^+$ 18.5%<br>$[M+Na]^+$ 33%<br>$[M+K]^+$ 100%	$[M+H]^+$ 73%<br>$[M+Na]^+$ 100%<br>$[M+K]^+$ 32%<br>$[M+CaNO_3]^+$ 89%
Sr^{2+}	$[M+H]^+$ 48%<br>$[M+Na]^+$ 100%<br>$[M+K]^+$ 18%<br>$[M+Sr-H]^+$ 23%	$[M+H]^+$ 34%<br>$[M+Na]^+$ 31%<br>$[M+K]^+$ 5%<br>$[M+Sr-H]^+$ 100%	$[M+H]^+$ 21%<br>$[M+Na]^+$ 4%<br>$[M+K]^+$ 22%<br>$[M+Sr-H]^+$ 100%	$[M+Na]^+$ 100%<br>$[M+K]^+$ 1%	$[M+H]^+$ 36%<br>$[M+Na]^+$ 100%<br>$[M+K]^+$ 91%	$[M+H]^+$ 74%<br>$[M+Na]^+$ 100%<br>$[M+K]^+$ 32%
Ba^{2+}	$[M+H]^+$ 20%<br>$[M+Na]^+$ 100%<br>$[M+K]^+$ 49%<br>$[M+Ba-H]^+$ 4.5%	$[M+H]^+$ 6%<br>$[M+Na]^+$ 100%<br>$[M+K]^+$ 15%<br>$[M+Ba-H]^+$ 10%	$[M+H]^+$ 14%<br>$[M+Na]^+$ 100%<br>$[M+K]^+$ 88%<br>$[M+Ba-H]^+$ 89%	$[M+Na]^+$ 100%	$[M+Na]^+$ 55.5%<br>$[M+K]^+$ 100%	$[M+H]^+$ 14%<br>$[M+Na]^+$ 100%<br>$[M+K]^+$ 89.5%
Pb^{2+}	$[M+H]^+$ 21%<br>$[M+Na]^+$ 100%<br>$[M+K]^+$ 18%<br>$[M+Pb-H]^+$ 67%	$[M+H]^+$ 4%<br>$[M+Na]^+$ 78%<br>$[M+K]^+$ 46%<br>$[M+Pb-H]^+$ 100%	$[M+H]^+$ 4%<br>$[M+Na]^+$ 57%<br>$[M+K]^+$ 18%<br>$[M+Pb-H]^+$ 100%	$[M+Na]^+$ 100%<br>$[M+K]^+$ 1%	$[M+Na]^+$ 92%<br>$[M+K]^+$ 100%	$[M+H]^+$ 10.5%<br>$[M+Na]^+$ 100%<br>$[M+K]^+$ 33%
Cu^{2+}	$[M+H]^+$ 100%<br>$[M+Na]^+$ 11%<br>$[M+K]^+$ 35%<br>$[M+Cu]^+$ 22%	$[M+H]^+$ 99%<br>$[M+Na]^+$ 100%<br>$[M+K]^+$ 31%<br>$[M+Cu]^+$ 37%	$[M+H]^+$ 9%<br>$[M+Na]^+$ 94%<br>$[M+K]^+$ 57%<br>$[M+Cu]^+$ 100%<br>$[M+2Cu-H]^+$ 10%	$[M+Na]^+$ 5.5%<br>$[M+Cu]^+$ 100%	$[M+Na]^+$ 6%<br>$[M+K]^+$ 100%<br>$[M+Cu]^+$ 33%	$[M+Cu]^+$ 100%
Ag^+	$[M+Ag]^+$ 100%<br>$[M+2Ag-H]^+$ 1%	$[M+Na]^+$ 2.6%<br>$[M+Ag]^+$ 100%<br>$[M+2Ag-H]^+$ 8%<br>$[M+3Ag-2H]^+$ <1%	$[M+Ag]^+$ 100%<br>$[M+2Ag-H]^+$ 6%<br>$[M+3Ag-2H]^+$ <1%	$[M+Ag]^+$ 100%	$[M+Na]^+$ 2.7%<br>$[M+K]^+$ 20%<br>$[M+Ag]^+$ 100%	$[M+Na]^+$ <1%<br>$[M+K]^+$ <1%<br>$[M+Ag]^+$ 100%
Eu^{3+}	$[M+H]^+$ 47%<br>$[M+Na]^+$ 18%<br>$[M+K]^+$ 12%<br>$[M+Eu-2H]^+$ 100%	$[M+Na]^+$ 9%<br>$[M+Eu-2H]^+$ 100%<br>$[M+EuNO_3-H]^+$ 2.8%	$[M+H]^+$ 100%<br>$[M+Na]^+$ 32%<br>$[M+K]^+$ 79%<br>$[M+Eu-2H]^+$ 69%	$[M+Na]^+$ 100%	$[M+H]^+$ 5%<br>$[M+Na]^+$ 47%<br>$[M+K]^+$ 100%<br>$[M+EuNO_3-H]^+$ 4.3%	$[M+H]^+$ 100%<br>$[M+Na]^+$ 53%<br>$[M+K]^+$ 32%<br>$[M+EuNO_3-H]^+$ 85%
Tb^{3+}	$[M+H]^+$ 88%<br>$[M+Na]^+$ 3.9%<br>$[M+K]^+$ 11%<br>$[M+Tb-2H]^+$ 100%	$[M+H]^+$ 20%<br>$[M+Na]^+$ 100%<br>$[M+K]^+$ 9%<br>$[M+Tb-2H]^+$ 70%	$[M+H]^+$ 100%<br>$[M+Na]^+$ 58%<br>$[M+K]^+$ 79%<br>$[M+Tb-2H]^+$ 96%	$[M+Na]^+$ 100%	$[M+Na]^+$ 8.4%<br>$[M+K]^+$ 100%	$[M+H]^+$ 100%<br>$[M+Na]^+$ 41%<br>$[M+K]^+$ 45%
Gd^{3+}	$[M+H]^+$ 27%<br>$[M+Na]^+$ 10%<br>$[M+Gd-2H]^+$ 100%	$[M+H]^+$ 8%<br>$[M+Na]^+$ 98%<br>$[M+K]^+$ 9%<br>$[M+Gd-2H]^+$ 100%	$[M+H]^+$ 35%<br>$[M+Na]^+$ 43%<br>$[M+K]^+$ 27%<br>$[M+Gd-2H]^+$ 100%	$[M+Na]^+$ 100%	$[M+H]^+$ 1.6%<br>$[M+Na]^+$ 24%<br>$[M+K]^+$ 100%	$[M+H]^+$ 34%<br>$[M+Na]^+$ 72%<br>$[M+K]^+$ 100%

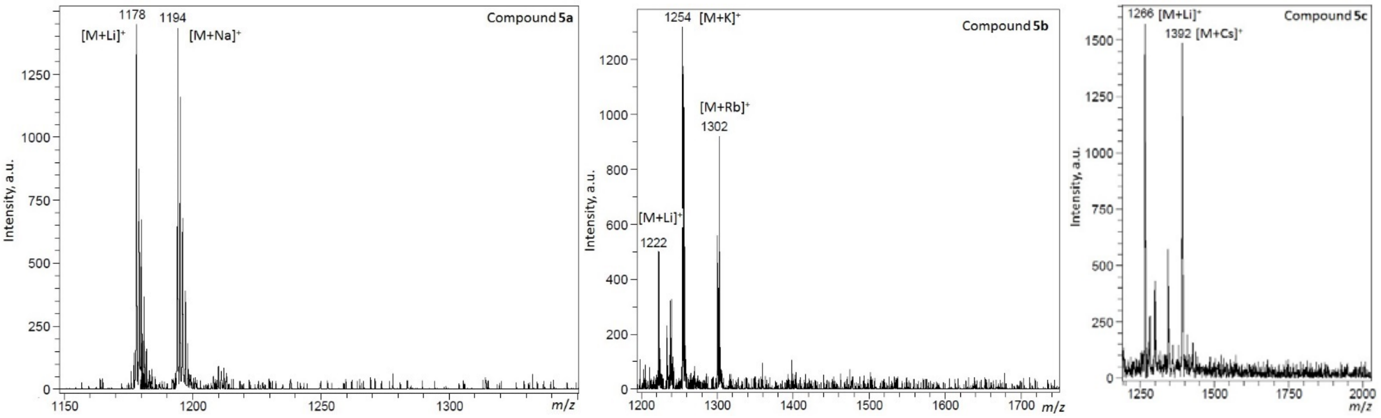

Fig. S22. MALDI mass spectra of the ligands **5a–c** mixed with metal nitrate salts.

Dynamic light scattering data of thiacalixcrown receptors

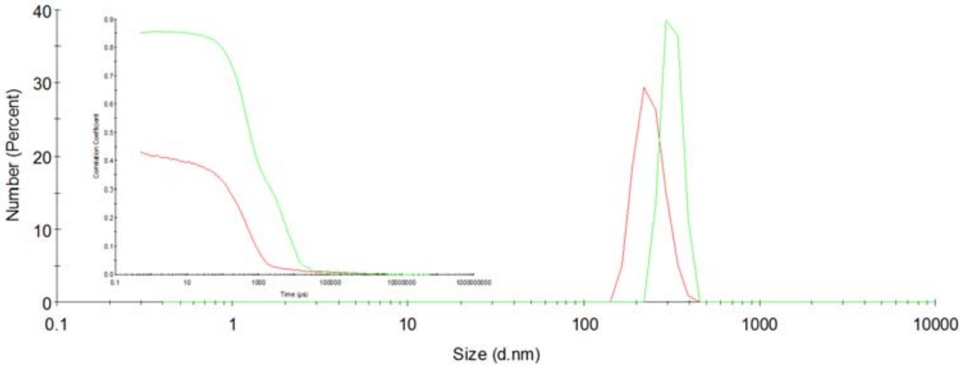

Fig. S23. Number-averaged particle size distribution plots and corresponding correlation functions of compound **2c** in organic phase (green line) and aqueous phase (red line) after extraction of $CsNO_3$.

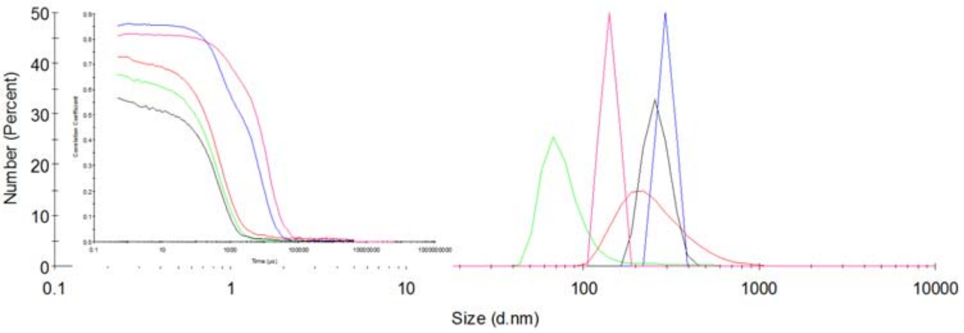

Fig. S24. Number-averaged PSD and corresponding correlation functions of compound **5c** in organic phase after mixing CH_2Cl_2 and water solvents (blue line); organic phase after extraction of CsPic (red line) and $CsNO_3$ (pink line); and aqueous phase after extraction of cesium picrate (green line) and cesium nitrate (black line).

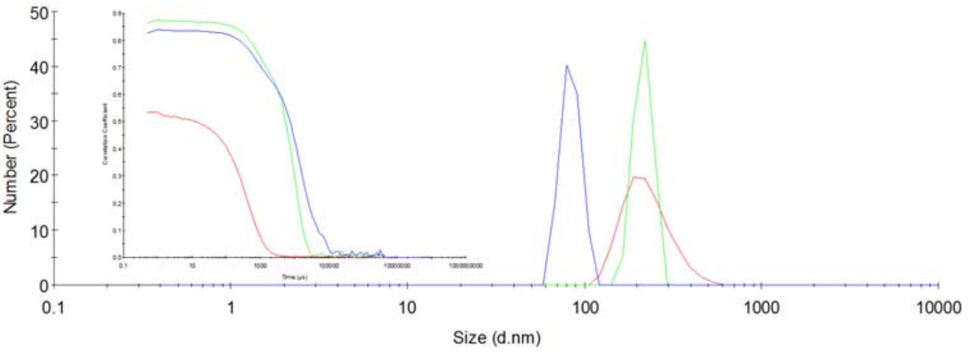

Fig. S25. Number-averaged PSD and corresponding correlation functions of compound **6c** in aqueous phase after mixing CH_2Cl_2 and water (red line) and organic phase after extraction of CsPic (blue line) and $CsNO_3$ (green line).

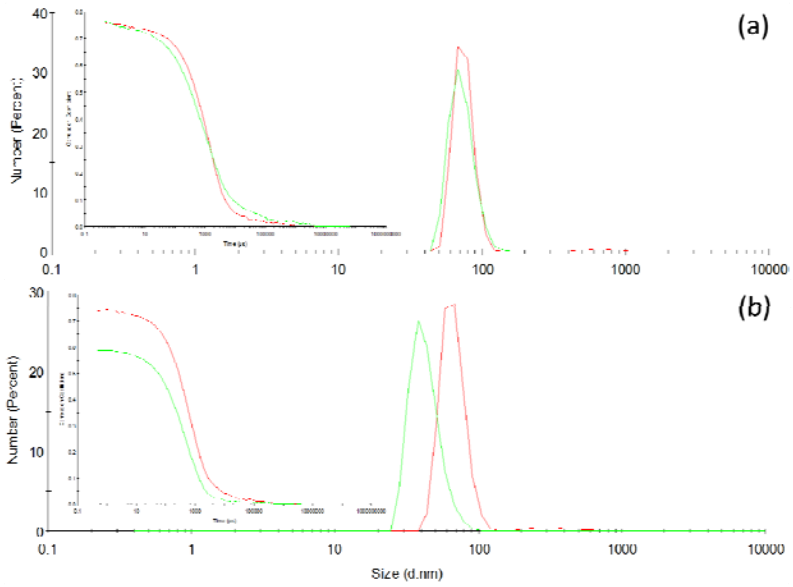

Fig. S26. Number-averaged PSD and corresponding correlation functions of compound (a) **2c** and (b) **5c** in aqueous phase after extraction of NaPic (red line) and RbPic (green line).

Computational data of thiacalix[4]monocrown-ethers

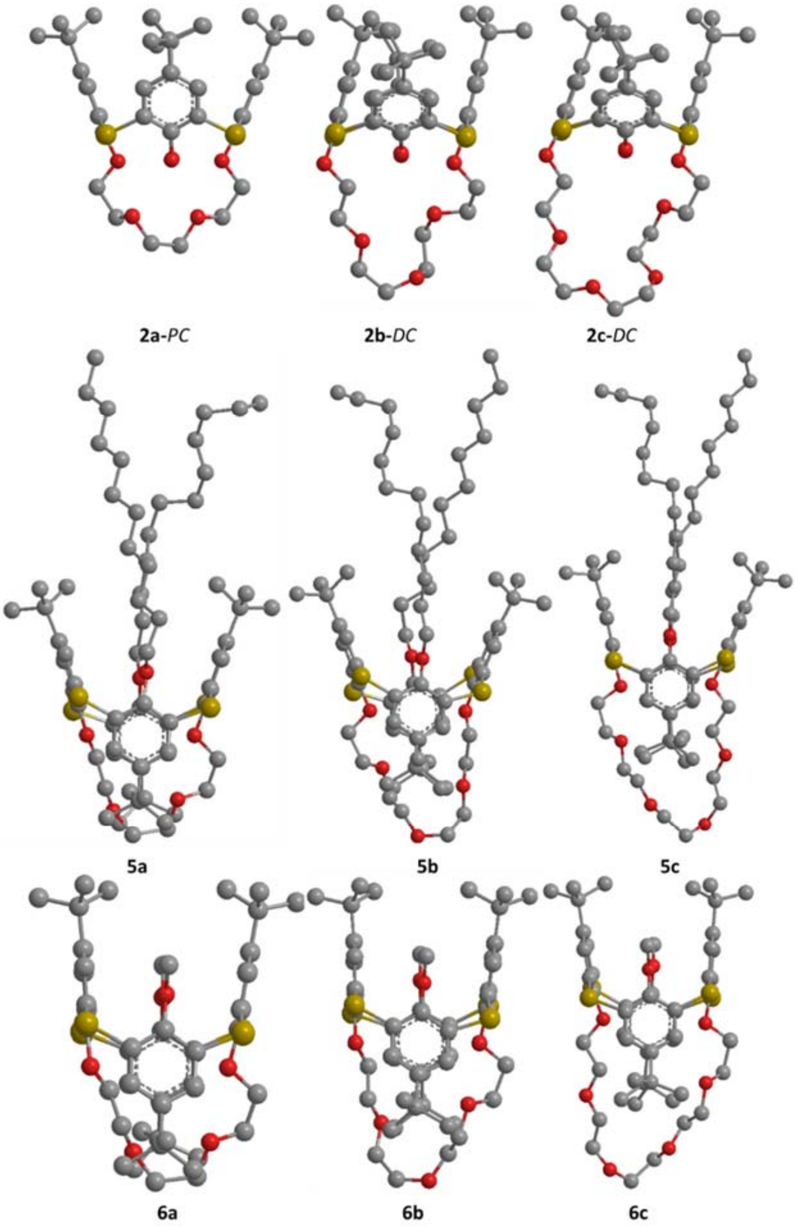

Fig. S27. MM+ minimized geometries of *PC*/*DC* stereoisomeric forms of ligands **2a**–**c** and *1,3-alternate* **5a–c** and **6a–c**.

Table S2. PBE/3z-calculated bond length values, Å, in lithium complexes of 12-crown-4 and compounds **2a** and **6a**.

Chemical bond	Compound		
	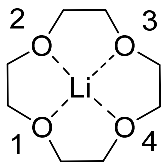	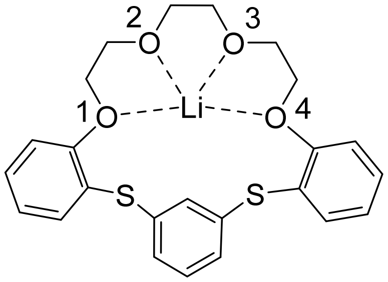 **6a**	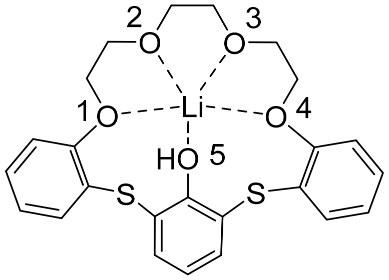 **2a**
Li–O1	1.95	2.14	1.95
Li–O2	2.02	2.16	2.01
Li–O3	2.01	2.09	2.40
Li–O4	2.03	2.45	3.29
O1–O4	2.68	4.01	4.60
O1–O2	2.68	2.70	2.62
Li–O5	–	–	1.93

Langmuir monolayer measurements of thiacalix[4]crown-ethers

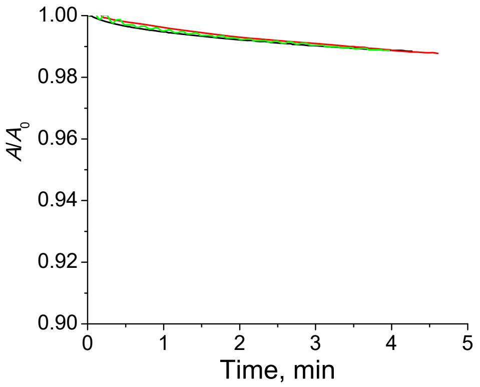

Fig. S28. Evolution of the mean molecular area of compounds **2a** (black, π = 11 mN/m), **2b** (red, π = 15 mN/m), and **2c** (green, π = 20 mN/m) in monolayers at constant surface pressure.

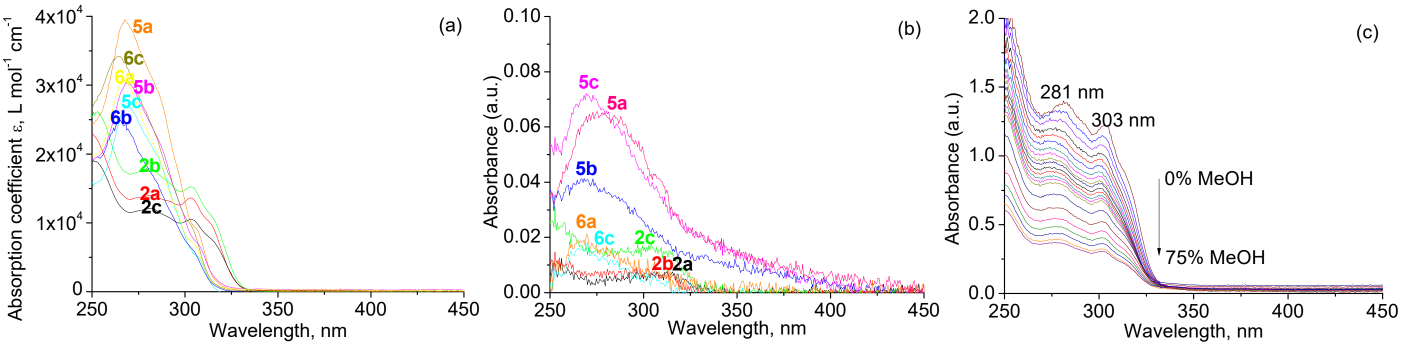

Fig. S29. (a) Electronic absorption spectra of compounds **2**, **5**, and **6** in $CHCl_3$ (c = (1.0–5.0) × 10^{-5} mol L^{-1}). (b) UVRAS spectra of compounds **2**, **5**, and **6** at the air–water interface (c = 0.1 mM (compounds **2** and **5**) and 0.01 mM (compounds **6**) in $CHCl_3$). (c) Electronic absorption spectra of **2c** in CH_2Cl_2–(0–3)MeOH. c_0 = 1.0 × 10^{-4} mol L^{-1}.

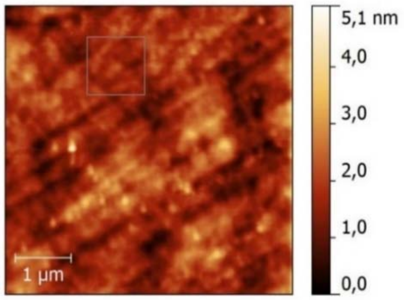

Fig. S30. Height image of bare quartz substrate (AFM, tapping mode, 5 μm × 5 μm).

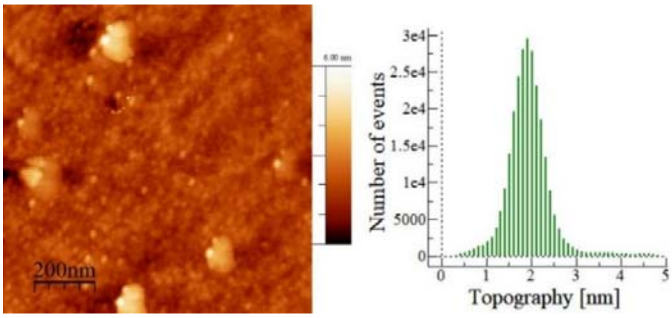

Fig. S31. Height image of quartz substrate with 1 monolayer of thiacalixcrown **2a** transferred at surface pressure of 10 mN/m (AFM, tapping mode, 1 μm × 1 μm) and the histogram corresponding to topography.

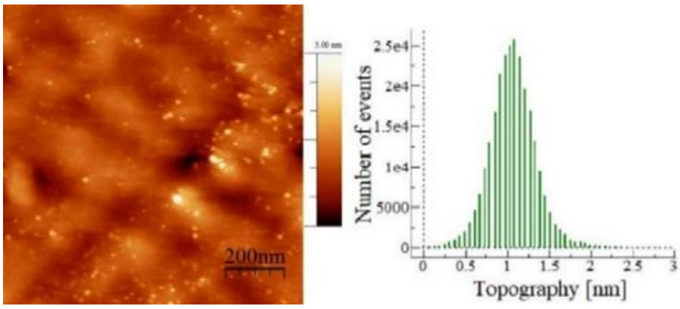

Fig. S32. Height image of quartz substrate with 1 monolayer of thiacalixcrown **2b** transferred at surface pressure of 12 mN/m (AFM, tapping mode, 1 μm × 1 μm) and the histogram corresponding to topography.

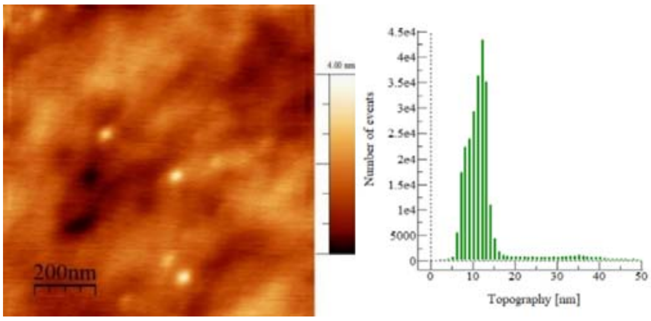

Fig. S33. Height image of quartz substrate with 1 monolayer of thiacalixcrown **5a** transferred at surface pressure of 6 mN/m (AFM, tapping mode, 1 μm × 1 μm) and the histogram corresponding to topography.

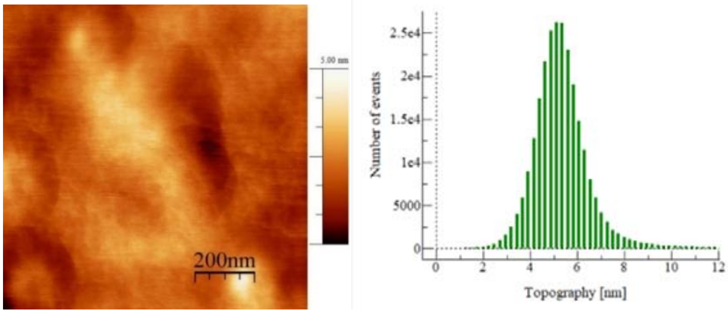

Fig. S34. Height image of quartz substrate with 1 monolayer of thiacalixcrown **5b** transferred at surface pressure of 6 mN/m (AFM, tapping mode, 1 μm × 1 μm) and the histogram corresponding to topography.

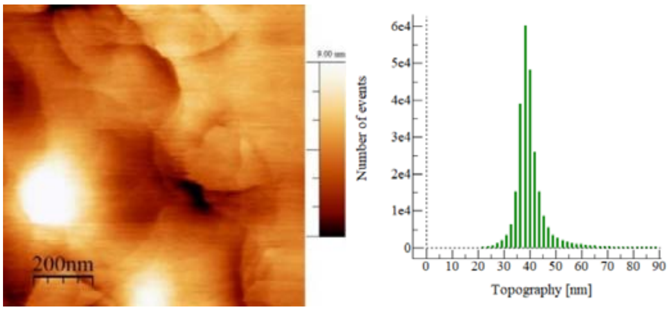

Fig. S35. Height image of quartz substrate with 1 monolayer of thiacalixcrown **6a** transferred at surface pressure of 10 mN/m (AFM, tapping mode, 1 μm × 1 μm) and the histogram corresponding to topography.

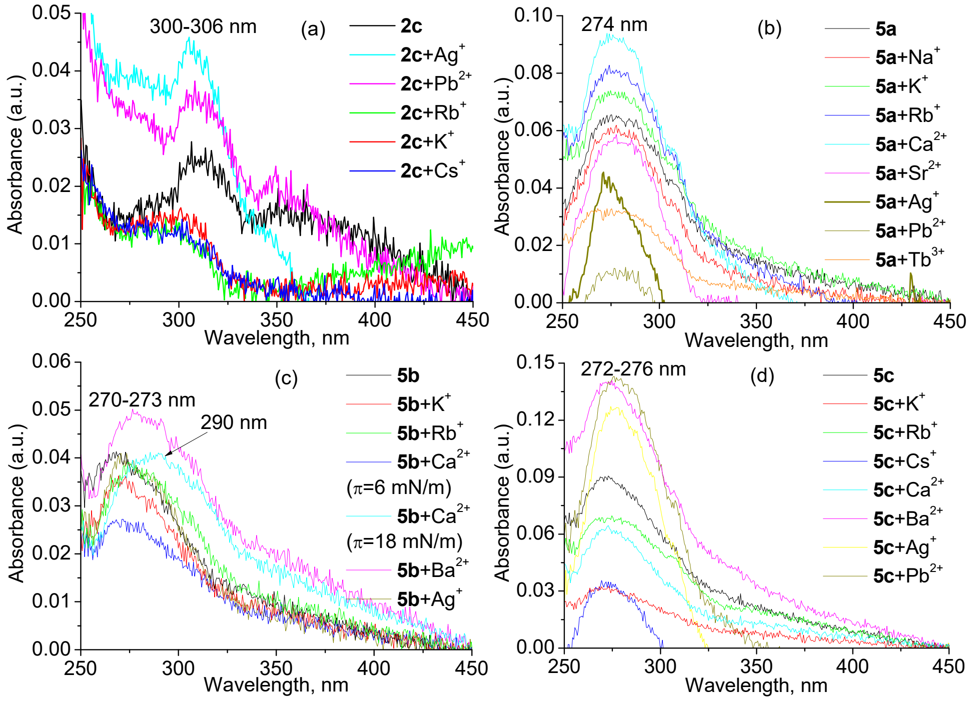

Fig. S36. UVRAS spectra of (a) **2c** and (b–d) **5a–c** at the air–0.01 M salt water interface (c = 0.1 mM in $CHCl_3$).

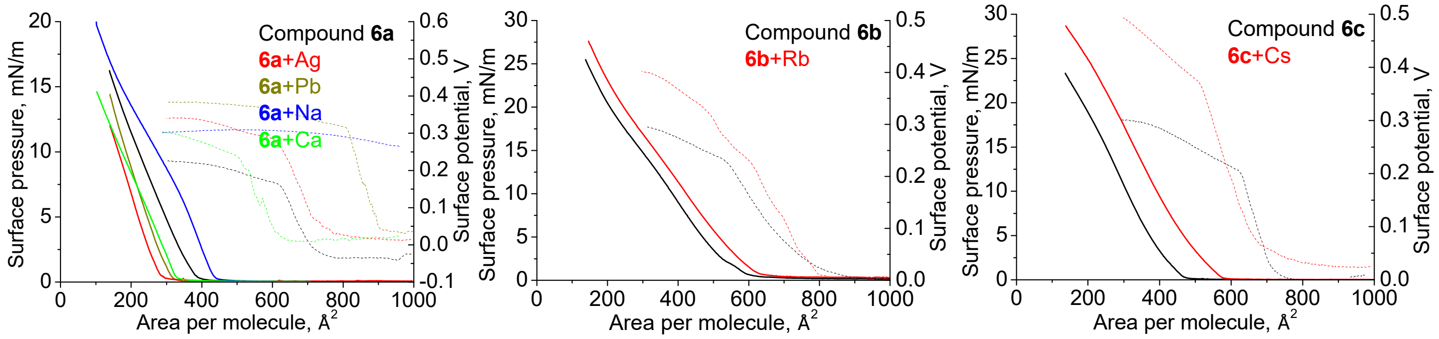

Fig. S37. Surface pressure/SPOT–molecular area isotherms of monolayers of thiacalixcrowns **6a–c** on water or 0.01 M salt water subphase. Concentration of the ligands in spreading solvent is 1 × 10^{-5} M.

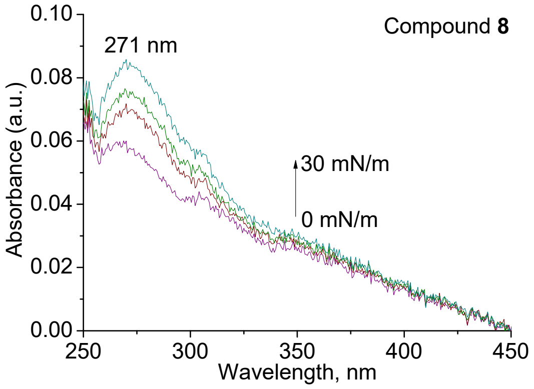

Fig. S38. UVRAS spectra of compound **8** at the air–water interface over monolayer compression (c = 0.1 mM in $CHCl_3$).

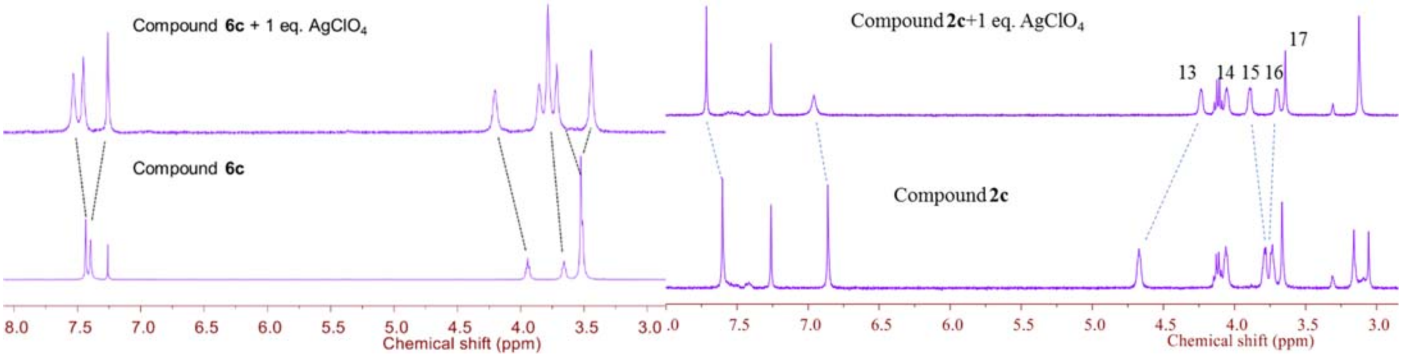

Fig. S39. Evolution of ^{1}H NMR spectra of compounds **6c** and **2c** on exposure to $AgClO_4$ in $CDCl_3$ (**6c**) and $CDCl_3$: CD_3OD (10:1) (**2c**) (numbering of protons is given in Scheme S1).

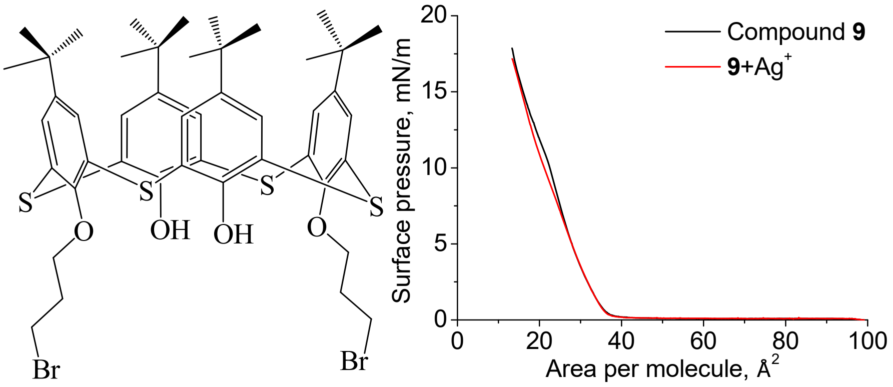

Fig. S40. The π–A isotherms of compound **9** on water or 0.01 M $AgNO_3$ subphase. $c = 1 \times 10^{-4}$ M in $CHCl_3$.

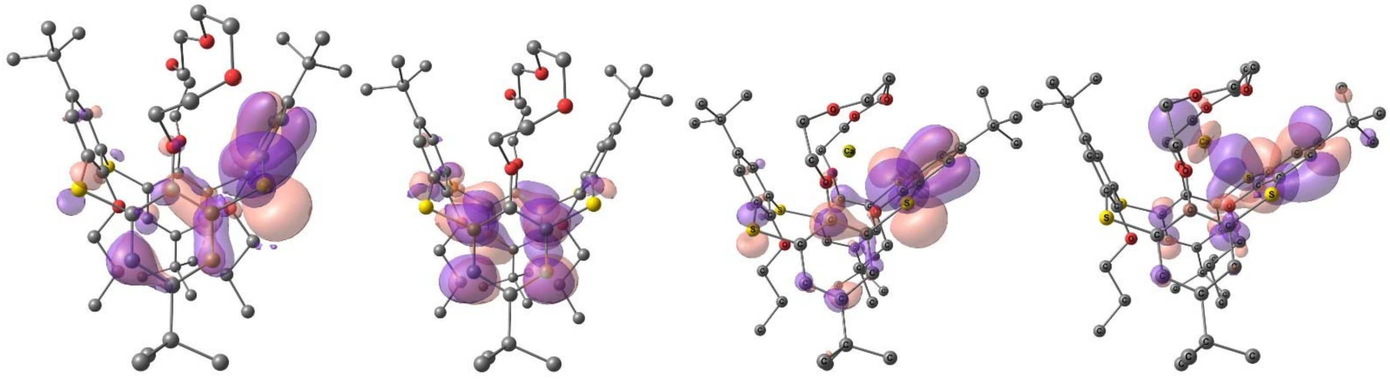

(a) (b) (c) (d)

Fig. S41. Frontier molecular orbitals contributing to low-energy absorption bands: a) **5b'** HOMO, b) **5b'** LUMO + 1, c) **5b'**–Ca HOMO-2, d) **5b'**–Ca LUMO + 1,
